# Aperiodic nanoplasmonic devices for directional colour filtering and sensing

**DOI:** 10.1038/s41467-017-01268-y

**Published:** 2017-11-07

**Authors:** Matthew S. Davis, Wenqi Zhu, Ting Xu, Jay K. Lee, Henri J. Lezec, Amit Agrawal

**Affiliations:** 1 Center for Nanoscale Science and Technology, National Institute of Standards and Technology, Gaithersburg, MD 20899 USA; 2 Maryland NanoCenter, University of Maryland, College Park, MD 20742 USA; 3 Department of Electrical Engineering and Computer Science, Syracuse University, Syracuse, NY 13244 USA; 4 National Laboratory of Solid State Microstructures, College of Engineering and Applied Sciences, and Collaborative Innovation Center of Advanced Microstructures, Nanjing University, Nanjing, 210093 China

## Abstract

Exploiting the wave-nature of light in its simplest form, periodic architectures have enabled a panoply of tunable optical devices with the ability to perform useful functions such as filtering, spectroscopy, and multiplexing. Here, we remove the constraint of structural periodicity to enhance, simultaneously, the performance and functionality of passive plasmonic devices operating at optical frequencies. By using a physically intuitive, first-order interference model of plasmon-light interactions, we demonstrate a simple and efficient route towards designing devices with flexible, multi-spectral optical response, fundamentally not achievable using periodic architectures. Leveraging this approach, we experimentally implement ultra-compact directional light-filters and colour-sorters exhibiting angle- or spectrally-tunable optical responses with high contrast, and low spectral or spatial crosstalk. Expanding the potential of aperiodic systems to implement tailored spectral and angular responses, these results hint at promising applications in solar-energy harvesting, optical signal multiplexing, and integrated sensing.

## Abstract (web summary)

Devices based on periodic architectures are intrinsically limited in terms of spectral response. Using an interference-based model, the authors design and fabricate plasmonic aperiodic groove array devices which function as light filters and colour sorters with flexible, multi-spectral optical response.

## Introduction

Scattering and interference phenomena govern the novel, and sometimes unexpected, physics associated with aperiodic optical systems that include weakly disordered, deterministic aperiodic, quasiperiodic, and random structures^1–5^. While the underlying mechanisms governing localization and wave-transport in these devices require further elucidation, remarkable progress has occurred in areas such as random lasing and imaging^6–8^. In recent years, plasmonic systems, utilizing electromagnetic waves that are confined to a metal-dielectric interface, have allowed confinement and manipulation of light on length scales that are simply not possible with purely dielectric systems^9–12^. For example, periodic arrays of metallic and metallo-dielectric scatterers patterned on a deep-subwavelength scale, commonly referred to as metasurfaces, have demonstrated abrupt changes to the phase-front of light allowing complex wavefront shaping using flat-optical components of nanoscale thickness^13,14^. One class of such structures, a periodic array of nanoscale apertures, slits or slit and grooves patterned on an opaque metal film, has shown promise as an efficient wavelength-scale transmission light-filter^15–17^ and chemical/biological sensor^18–21^. The underlying periodicity inherent to these structures allows a wide-range of analytical methods to be used for device-design^22–25^. However, since periodicity a-priori limits the range of possible spectral responses, devices based on periodic structures are intrinsically limited in their functional characteristics. In comparison, aperiodic structures are less constrained in their configuration both in real and reciprocal space, and therefore potentially allow greater engineering control over the optical response of devices which incorporate them^26,27^. A large variety of recently demonstrated aperiodic structures have added significant flexibility and richness towards engineering an optical response in ways not possible with periodic counterparts. For example, computationally intensive nonlinear search algorithms were employed to design ultra-compact polarization beam splitters and wavelength demultiplexers at telecom wavelengths, wherein the algorithm searched the full design-space of the device area with arbitrary topologies for the optimum solution^28,29^. Alternate approaches utilizing the transfer matrix method^30^, aperiodic Fourier modal method^31,32^, and field-decomposition^33^, or using asymmetric device profiles^34^ have recently been used to predict the scattering properties of surface plasmon polaritons (SPPs) from subwavelength patterns on a metal surface, and were utilized to achieve dichroic beam splitting^35^ and directional launching of SPPs for normally incident light at a single wavelength of interest^34,36,37^. However, the widespread use of aperiodic structures in optical devices has been inhibited to date by the constraints of computationally intensive optimization based on multi-dimensional parameter searches using full electromagnetic numerical simulations^28,29,38^.

Here, we show how the use of a physically intuitive, first-order interference model of plasmon-light interactions enables straightforward design of aperiodic plasmonic devices with flexible and angle-dependent multi-spectral transmission signatures. Following this approach, we experimentally implement visible frequency transmission filters that leverage an aperiodic arrangement of metallic surface grooves to yield unique spectral and angular responses, in which a discrete set of input (or output) angles is mapped one-to-one to a discrete set of output (or input) frequencies. The device consists of a single-subwavelength linear slit (circular aperture) surrounded by multiple linear (annular) grooves on an opaque metal film with the position, width and depth of the grooves individually optimized to achieve the desired multi-spectral response at specific incident angles. The structure is designed using a nested iterative search algorithm based on a physically intuitive first-order analytic model of interference, at the slit, between directly incident light and SPPs arriving from the illuminated grooves, each acting as SPP launch sites. Use of an aperiodic arrangement for groove placement with respect to the slit affords utmost flexibility in tailoring the spectral response of the device at arbitrary angles of incidence and over a broad spectral range simultaneously. The interference model is physically intuitive and vastly simplified compared to full numerical simulations typically underlying nonlinear search algorithms because it requires only knowledge of SPP coupling and phase-shift coefficients for optimum structure design. The deployment here of a first-order analytical model as the core of a numerical optimization algorithm serves to confirm that the fundamental interference mechanisms, shown to govern operation of periodic slit-groove devices implemented to date, can also be successfully applied to the more general case of aperiodic plasmonic systems provided that other geometrical degrees of freedom are also enabled. This approach, involving only adjustment of in-plane dimensions (individual groove spacing and width), results in an easy-to-fabricate device having a complex multi-functional response at optical frequencies. Furthermore, we demonstrate that the model is broadly applicable by utilizing it to minimize the transmission spectral linewidth for a refractive index sensing application. Expanding the potential of aperiodic systems to implement tailored optical responses, these results hint at potential applications in hyperspectral imaging, multi-junction photovoltaics and integrated sensing.

## Results

### First-order interference model for SPP-light interactions

**Fig. 1:**
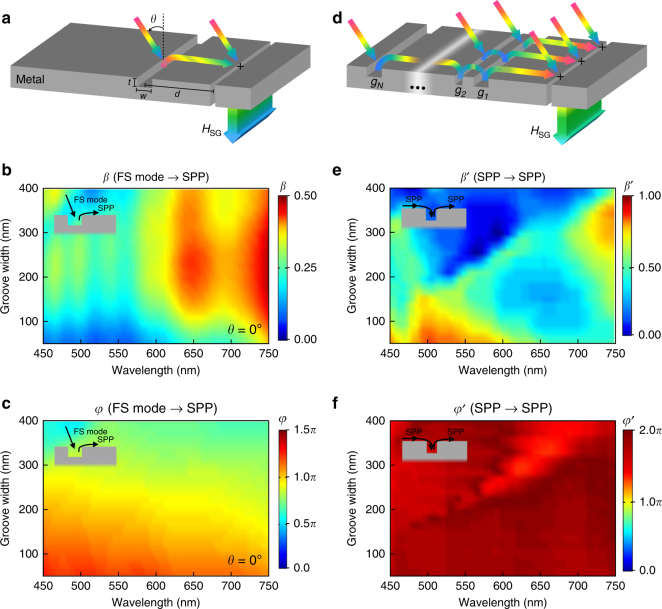
Transmission mechanism through an aperiodic slit-groove device. **a** *H*-field amplitude transmission from a slit in the presence of a single-groove placed to its left, illuminated by a TM polarized white light laser (*H*-field parallel to the slit-length) at an angle *θ* on a metal-dielectric interface, where *d* is the distance between the slit and the groove (of width *w* and depth *t*). The SPP coupling process at the location of the groove is characterized by a real coupling coefficient *β* and phase *φ*. **b**, **c** Variation in *β* and *φ* as a function of both groove-width (*w*: 50–400 nm) and free-space wavelength (*λ*_0_: 450–750 nm), for illumination at normal incidence (*θ* = 0°) and fixed groove-depth (*t* = 100 nm) at a Ag-air interface. **d** *H*-field amplitude transmission through a device consisting of *N* grooves located to the left of an isolated slit. The propagation of an SPP coupled from an arbitrary groove *g*_*N*_ propagating over an intermediate groove on its way to the slit undergoes a phase-shift *φ*′ and an amplitude reduction of a factor *β*′. In both **a**, **d** the superimposed guided-mode propagation, through the slit, of both the free-space light and the SPP is characterized by a relative *H*-field coupling coefficient B_eff_ and phase-shift *Φ*_eff_ (Supplementary Fig. 2). **e**, **f** Variation in *β*′ and *φ*′ as a function of groove-width (*w*: 50–400 nm) of an intermediate groove, and free-space wavelength (*λ*_0_: 450–750 nm), and fixed groove-depth (*t* = 100 nm) at a Ag-air interface

The spectral transmission response of an SPP-based slit-groove device can be explained by first analyzing the angle-dependent transmission through an interferometer consisting of an opaque metal film, facing a dielectric medium of refractive index *n*, and decorated with both a subwavelength-width through-slit (width: *W*) and a parallel subwavelength-width groove (width: *w*; depth: *t*) placed to the left of the slit at a center-to-center distance *d* (Fig. 1a). The film is illuminated on the groove side, at an angle *θ* with respect to the surface normal, by a transverse-magnetic (TM) polarized plane-wave (free-space (FS) wavelength: *λ*_0_; wavevector magnitude: *k*_0_), with *H*-field parallel to the slit. *H*_0_ designates the complex *H*-field amplitude of the incident wave at the slit; the corresponding *H*-field amplitude of the incident wave at the groove is then ${H_0}{e^{i\phi }}$, where $\phi = - n{k_0}d\,{\rm{sin}}\,\theta$ is the phase retardation of the plane-wave at the groove with respect to the slit. The incident light at the groove diffracts into an SPP mode of field amplitude ${H_0}{e^{i\phi }}\beta {e^{i\varphi }}$, and mode index ${n_{{\rm{SPP}}}} + i{\kappa _{{\rm{SPP}}}}$, where real coefficients $\beta \left( {w,t,\theta ,{\lambda _0}} \right)$ and $\varphi \left( {w,t,\theta ,{\lambda _0}} \right)$ represent the amplitude and phase of the FS to SPP coupling process (assumed to depend on groove width and depth as well as wavelength and angle of the incident light). The SPP propagates toward the slit, where it arrives with complex field amplitude ${H_0}{e^{i\phi }}\beta {e^{i\varphi }}{e^{i\psi }}{e^{ - \left( {d/\alpha } \right)}}$, where $\psi = {n_{{\rm{SPP}}}}{k_0}d$ and $\alpha = 1{\rm{/}}\left( {{\kappa _{{\rm{SPP}}}}{k_0}} \right)$ are, respectively, the accumulated propagation phase and amplitude decay-length of the SPP along the surface. Finally, FS and SPP modes incident upon the slit are converted into coherently superimposed guided modes inside the slit, with amplitude coupling coefficient B(*W*, *θ*, *λ*_0_) and phase shift ${{\Phi }}\left( {W,\theta ,{\lambda _0}} \right)$ for the plane wave incident upon the slit from FS (where coupling is assumed to depend on slit width as well as both wavelength and incident angle), and coupling coefficient ${\rm B}\prime \left( {W,{\lambda _0}} \right)$ and phase-shift ${{\Phi }}\prime \left( {W,{\lambda _0}} \right)$ for the SPP incident upon the slit along the metal interface (where coupling is assumed to depend only on the slit width and FS wavelength). The net complex field amplitude at the output plane of the slit is then given by ${H_{{\rm{SG}}}} = T\left( {{H_0}B{e^{i{{\Phi }}}} + {H_0}{e^{i\phi }}\beta {e^{i\varphi }}{e^{i\psi }}{e^{ - \left( {d/\alpha } \right)}}B'{e^{i{{\Phi} {'}}}}} \right)$, where *T* is the complex amplitude transmission coefficient of the slit. For reference, the complex field amplitude at the output plane of an isolated slit, illuminated under identical conditions, is given by ${H_{\rm{S}}} = T{H_0}B{e^{i{{\Phi }}}}$. The groove-slit interference process then yields a net complex field amplitude at the output plane of the groove-decorated slit, relative to that of an isolated slit, given by

1 $$\gamma = \frac{{{H_{{\rm{SG}}}}}}{{{H_{\rm{S}}}}} = 1 + {B_{{\rm{eff}}}}{e^{i{{{\Phi }}_{{\rm{eff}}}}}}\beta {e^{ - \left( {d/\alpha } \right)}}{e^{i\left( {\varphi + \psi - \phi } \right)}},$$

where quantities ${B_{{\rm{eff}}}}\left( {W,\theta ,{\lambda _0}} \right) = B'\left( {W,{\lambda _0}} \right){\rm{/}}B\left( {W,\theta ,{\lambda _0}} \right)$ and ${{{\Phi }}_{{\rm{eff}}}}\left( {W,\theta ,{\lambda _0}} \right) = {{\Phi} {'}}\left( {W,{\lambda _0}} \right) - {{\Phi }}\left( {W,\theta ,{\lambda _0}} \right)$ represent, respectively, the amplitude and phase of the SPP-to-slit in-coupling process normalized to the amplitude and phase of the FS-to-slit in-coupling process. The corresponding transmitted intensity into the far-field for the slit-groove device, relative to that of an isolated slit, is then given by ${{\Gamma }} = {\left| \gamma \right|^2}$. The values of *β* and *φ* are calculated, in the case of a single slit-groove pair device patterned in a 250-nm-thick Ag-film facing FS, by curve-fitting the analytical expression for relative transmission intensity, *Γ*, to its value derived from two-dimensional finite-difference-time-domain (FDTD) simulations. The variations in *β* and *φ* as a function of both groove-width (*w*: 50–400 nm) and FS wavelength (*λ*_0_: 450–750 nm) for illumination at normal incidence (*θ* = 0°) and fixed groove-depth (*t* = 100 nm) are shown in Fig. 1b, c, respectively. The corresponding plots for the variations in *β* and *φ* as a function of *w* and *λ*_0_ for respective incident angles *θ* = 10° and 20° can be found in Supplementary Fig. 1. The variations in *B*_eff_ and *Φ*_eff_ as a function of FS wavelength (*λ*_0_: 450–750 nm), for illumination angles of *θ* = 0°, 10°and 20° and fixed slit-width (*W* = 100 nm) along with those in constituent parameters *B*, *B*′, *Φ*, and *Φ*′ are shown in Supplementary Fig. 2. Contributions from higher-order interference effects (such as multiple reflections of SPPs between the slit and the groove, or between grooves) are not taken into account as they are expected to be minimal^39^.

This approach can now be extended to model the transmission characteristics of a device consisting of *N* grooves located to the left of a single slit, each having arbitrary depth and width (Fig. 1d). Once again, the film is illuminated, at an angle *θ* with respect to the surface normal, with complex *H*-field amplitude *H*_*0*_ at the slit. The corresponding *H*-field amplitude of the incident wave at the groove *g*_*i*_ (located at a distance *d*_*i*_ from the slit) is then ${H_0}{e^{i{\phi _i}}}$, where ${\phi _i} = - n{k_0}{d_i}\,{\rm{sin}}\,\theta$ is the phase retardation of the plane-wave at the groove with respect to the slit. FS illumination of the groove results in launching of an SPP mode toward the slit with relative amplitude and phase *β*_*i*_ and *φ*_i_, respectively. Upon crossing any intermediate groove, *g*_*j*_ on its way to the slit, the SPP is modeled to undergo a phase-shift $\varphi _j^\prime$ and an amplitude reduction (modulation factor $\beta _j^\prime$) due to its interaction with that specific groove. In addition, the SPP experiences an accumulated propagation phase and amplitude decay-length along the surface of ${\psi _i} = {n_{{\rm{SPP}}}}{k_0}{d_i}$ and *α*, respectively. Upon arrival at the slit entrance, this SPP couples to the slit with amplitude coupling coefficient ${\rm B}'\left( {W,{\lambda _0}} \right)$ and phase-shift ${{\Phi} {'}}\left( {W,{\lambda _0}} \right)$, and coherently interferes with the waveguide modes resulting, from SPPs arriving from all the other grooves (and coupled into the slit with the same coupling coefficient and phase shift) and from direct illumination of the slit, with amplitude coupling coefficient ${\rm B}\left( {W,\theta ,{\lambda _0}} \right)$ and phase shift ${{\Phi }}\left( {W,\theta ,{\lambda _0}} \right)$. The net normalized *H*-field transmission amplitude relative to that of an isolated slit then given by

2 $${\gamma _N} = 1 + {{\rm B}_{{\rm{eff}}}}{e^{i{{{\Phi }}_{{\rm{eff}}}}}}\mathop {\sum }\limits_{i = 1}^N \left( {{\beta _i}{e^{ - \left( {{d_i}/\alpha } \right)}}{e^{i\left( {{\varphi _i} + {\psi _i} - {\phi _i}} \right)}}{{\left( {\mathop {\prod }\limits_{j = 1}^{i - 1} \beta _j^\prime {e^{i\varphi _j^\prime }}} \right)}_{i  >1}}} \right).$$

Generalizing the device to an aperiodic slit-groove structure having *N* grooves to the left and *M* grooves to the right of the slit, yields a normalized *H*-field transmission amplitude relative to that of an isolated slit of

3 $${\gamma _{MN}} = {\gamma _N} + {{\rm B}_{{\rm{eff}}}}{e^{i{{{\Phi }}_{{\rm {eff}}}}}}\mathop {\sum }\limits_{i = 1}^M \left( {{\beta _i}{e^{ - \left( {{d_i}/\alpha } \right)}}{e^{i\left( {{\varphi _i} + {\psi _i} + {\phi _i}} \right)}}{{\left( {\mathop {\prod }\limits_{j = 1}^{i - 1} \beta _j^\prime {e^{i\varphi _j^\prime }}} \right)}_{i  >1}}} \right).$$

The corresponding relative transmission intensity into the far field is given by ${{\Gamma }} = {\left| {{\gamma _{MN}}} \right|^2}$. FDTD simulations of a slit-groove device having two grooves (*N* = 2, *M* = 0) are used to derive the dependence groove-crossing amplitude-drop *β*′ and phase-slip *φ*′ on groove-profile and illumination wavelength. The variations in *β*′ and *φ*′ as a function of both groove-width (*w*: 50–400 nm) and FS wavelength (*λ*_0_: 450–750 nm), at fixed groove-depth (*t* = 100 nm), are shown in Fig. 1e, f, respectively. The first-order interference model of Eq. (3) forms the core of a multi-dimensional iterative optimization algorithm described as follows. Based on specifications for the model output, corresponding to desired characteristics for the transmitted intensity (such as spectral shape including discrete peak positions and linewidths), for given model inputs, corresponding to imposed illumination conditions (such as a range of FS wavelengths and angles of incidence), the algorithm performs a nested iterative-adaptive search in which the number of grooves and individual position, width and depth of each groove are simultaneously varied, using a least-square criterion to establish convergence.

### Aperiodic angle-selective colour filter

**Fig. 2:**
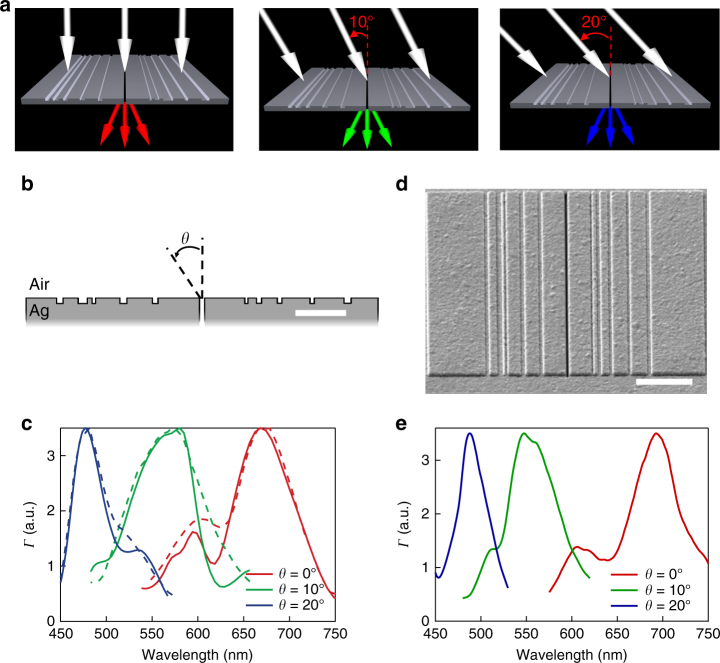
Aperiodic directional RGB colour-filter. **a** Schematic illustration of the angle-dependent light transmission characteristics of an angularly selective aperiodic RGB colour filter. The device is targeted to transmit red light at an incidence angle *θ* = 0°, green at *θ* = 10°, and blue at *θ* = 20° when illuminated with a TM polarized white-light laser source. The device consists of a subwavelength-width linear slit surrounded by five grooves each on both sides within a total lateral device dimension of ≤10 μm. **b** Model predicted surface cross-section profile of the optimized device at a Ag-air interface. Scale bar represents 1 μm. **c** Relative transmission (*Γ*) spectra calculated using the interference model of Eq. (3) (solid lines) and FDTD simulations (dashed lines) show remarkable agreement, and demonstrate the unique spectral transmission characteristics of the device. **d** Scanning-electron-microscope (SEM) image of the patterned surface of the aperiodic slit-groove array device taken at 38° from the surface normal. The device is fabricated using the procedure described in Methods section and outlined in Supplementary Fig. 6. Horizontal scale bar represents 2 μm. **e** Experimentally measured *Γ* spectra at three angles of incidence for the fabricated device. The spectral characteristics of the fabricated device, namely: linewidth, optical contrast, and spectral crosstalk, are summarized in Supplementary Tables 1 and 2

The algorithm described above provides an elegant platform for engineering the optical response of aperiodic slit-groove transmission devices, and suggests a broader range of applications than is possible with periodic arrays. For example, while interference filters and waveplates provide an easy route towards achieving high-contrast frequency and polarization selectivity, implementing optical frequency components that provide angular or directional selectivity over a broad spectral range represents a major technological challenge. Approaches utilizing anisotropic metamaterials and plasmonic slit-arrays have been proposed to achieve broadband angular selectivity, though at microwave frequencies^40,41^. A one-dimensional photonic crystal was recently used to achieve complete transparency over a broad spectral range at one incident angle^42^. Here, we first use the algorithm to design a plasmonic angularly selective colour filter that exhibits directionally modulated spectral output at optical frequencies under white-light plane-wave illumination. This filter yields tailored narrowband transmission spectra for specific incidence angles, where each spectral peak position (respectively in the red, green, and blue) of the transmitted light corresponds to one of three pre-defined incident angles (Fig. 2a). As an illustrative example, we target a structure that transmits light at center wavelengths of 690, 550, and 460 nm respectively, for incident angles of 0°, 10°, and 10°, respectively. Based on the above spectral excitation and angular transmission specifications, along with the respective design choices of: (a) Ag as the constituent metal film, (b) five grooves to each side of the slit (i.e., *N* = 5 and *M* = 5, limiting the device footprint to a lateral dimension of *L* ≤ 10 µm), and (c) a fixed groove-depth *t* = 100 nm, the search algorithm yields an optimized width and position for each groove relative to the slit. A schematic cross-section of the resulting aperiodic surface profile is shown in Fig. 2b. The model-calculated transmission spectra for the slit-groove structure relative to that of an isolated slit, *Γ* (Fig. 2c, solid lines), display a distinct spectral peak at each of the specified incidence angles (with red, blue, and green peak positions of 670, 545, and 476 nm, closely matching the respective target values). Each peak is characterized by low spectral crosstalk with respect to the two other peaks, as enforced by the search algorithm. The corresponding relative transmission spectra numerically simulated using the FDTD technique (Fig. 2c, dashed lines), where Ag-film thickness *h* = 250 nm and slit-width *W* = 100 nm are assumed, show remarkable agreement with the model spectra, validating in particular the efficacy of the assumptions underlying the first-order analytical model. The spectral peak locations resulting from the final aperiodic device design are also consistent with the associated spatial-frequency content denoted by discrete peaks in the reciprocal-space representation of the projection of the surface profile onto the plane of incidence, for each of the three angles of incidence (Supplementary Fig. 3). Aperiodic systems achieved using this approach differ from deterministic aperiodic geometries generated using substitution rules or self-similar inflation symmetries, and have been referred to as aperiodic systems by design in the literature^4,43–45^.

To further validate the efficacy of the aperiodic design, equivalent slit-groove devices where the grooves were arranged periodically or in a chirped (linear and exponential) geometry were designed for comparison (using constant groove width: *w* =50 nm and groove depth: *t* = 50 nm). As a basis for comparison with the aperiodic design, the periodic and chirped devices were designed to also exhibit a spectral peak in transmission at 690 nm under normal incidence illumination (Supplementary Fig. 4). For the other incidence angles of operation (*θ* = 10° and 20°), however, correct spectral positioning of the targeted transmission peaks was not possible: the chirped devices exhibit a complex transmission spectra with no specific trend, whereas the spectral peak in the periodic device shifts to shorter wavelengths that are determined by its spatial-frequency content (Supplementary Fig. 5). This comparison clearly illustrates that the underlying periodicity a-priori determines the spectral (or spatial) response of devices based on periodic architectures, and hence these devices cannot achieve the flexibility in engineering the optical response possible with aperiodic systems by design. Finally, random placement of the above grooves within the 10 µm-wide lateral footprint of the device yields a total absence of angular-spectral control, as evidenced by the transmission spectrum of one such random device (Supplementary Fig. 4d).

The groove-profile yielded by the optimization algorithm (Fig. 2b) was experimentally implemented (Fig. 2d) into a Ag-film on an indium-tin-oxide (ITO) coated fused silica substrate. The groove structure was defined using a sequential combination of electron-beam lithography and lift-off of an initial Ag-film (100-nm thick), followed by further evaporation of another Ag-film (150-nm thick). Focused-ion-beam (FIB) milling was then used to define a 100-nm wide, 10-µm long through slit (Methods section, and Supplementary Fig. 6 for fabrication details). A reference device consisting of an isolated 100-nm wide through slit on the same Ag-film was also fabricated by FIB milling. The spectral transmission characteristics of the device were measured by illuminating it with a TM-polarized supercontinuum white-light laser at three angles of incidence (0°, 10°, and 20°) with respect to the surface normal, with *H*-field parallel to the slit-length. The light transmitted through the device was collected using a ×100 microscope objective (numerical aperture (NA) = 0.75) and directed to a grating spectrometer coupled to a charge-coupled device (CCD) camera. The experimentally measured relative transmission spectra at each angle of incidence (normalized to that of the isolated reference slit) are shown in Fig. 2e. A close match to the model predictions were obtained, namely: distinct red, green, and blue spectral peaks, respectively, at each of the illumination angles; peak positions of 690, 545, and 480 nm, closely matching both target and analytic model-computed values; and low-crosstalk evidenced by low-transmittance out-of-band spectral features that match the analytic predictions. Non-optimized, full-width-at-half-maximum (${\rm{\Delta }}{\lambda _{1/2}}$) linewidth values for each of the peaks are 60 nm (*θ* = 0°), 60 nm (*θ* = 10°), and 38 nm (*θ* = 20°), respectively, which are systematically smaller by a factor of approximately two to four compared to those reported in the literature for plasmonic transmission devices incorporating periodic arrays of grooves^46–48^. The optical contrast, linewidth, and spectral crosstalk performance of the optimized angle-resolved colour sorter is further discussed in Supplementary Note 1 and Supplementary Tables 1 and 2. Moreover, mapping the angle of incident radiation to a given colour using optimized aperiodic groove positions can be readily extended to more than three input angles (e.g., five as shown in Supplementary Fig. 7). The complete summary of the aperiodic device implementation procedure including modeling and optimization, nanofabrication, and experimental characterization is outlined in Supplementary Fig. 8. Note that the absolute transmission efficiency of the aperiodic device at the spectral peak locations is on the order of 1.5–3 % across the visible frequency range for illumination with a TM polarized light. For reference, the FDTD-simulated absolute transmission efficiency of an isolated single-slit as a function of Ag-film thickness *h* and slit-width *W*, for a normally incident TM polarized light at three wavelengths (690, 550, and 460 nm) is shown in Supplementary Fig. 9.

### Anti-symmetric spatial spectrum splitting

**Fig. 3:**
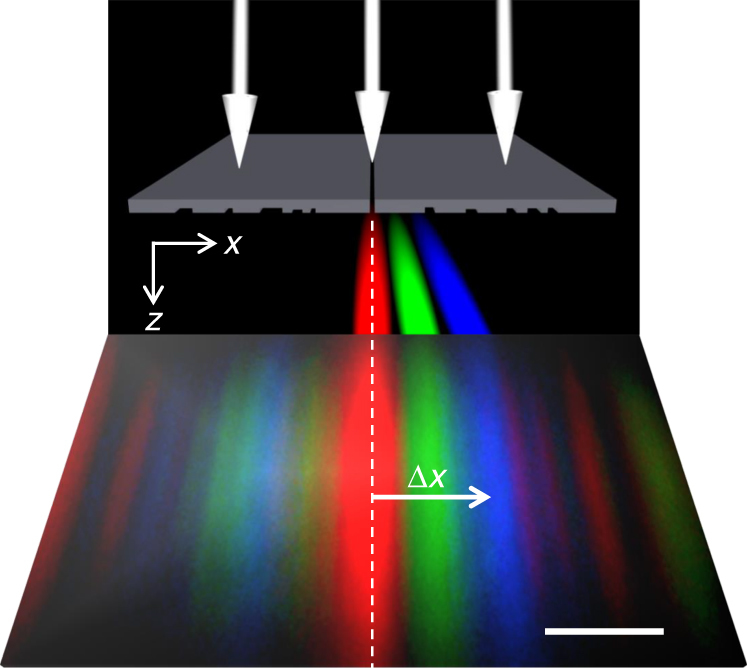
Spectrum-splitting using the aperiodic slit-groove device. top panel, Schematic illustration of the device transmission under “reverse illumination” from the non-patterned side. Owing to reciprocity, the device when illuminated with TM polarized white light laser source from the non-patterned side is able to spatially separate the three colours along well-defined discrete angles on the same side of the surface normal. bottom panel, Wide-field projected image of the transmitted light, collected at a distance Δ*z* = 17.5 μm from the exit surface of the device, using an inverted optical microscope (×100, NA = 0.75 microscope objective) connected to a colour-CCD camera. Scale bar along the *x*-axis in the CCD image represents 6.5 μm. The diffraction angle $\theta = {\rm{ta}}{{\rm{n}}^{ - 1}}\left( {{\rm{\Delta }}x{\rm{/\Delta }}z} \right)$ for the three colours is determined by directly measuring the distance of the local intensity maximum of the red, green, and blue streaks relative to the center of the slit, Δ*x*. The experimentally measured diffraction angles for the red, green, and blue colours (0° ± 0.49°, 9.72° ± 0.47°, and 18.92° ± 0.44°, respectively) match closely the angles specified for angle-selective colour-filter operation under “forward illumination” (0°, 10°, and 20°, respectively)

Spectrum splitting using diffractive optics has been utilized in recent years to enhance the photovoltaic output power in solar cells^49^ as well as for hyperspectral imaging applications^15,46,50^. Periodic plasmonic antennas have also been utilized to achieve symmetric, angularly continuous, directional spectral sorting of white-light^47^ or emission from quantum dots and fluorophores^51,52^. The angle-resolved colour sorter described above, on the other hand, can be exploited to achieve anti-symmetric spatial spectrum-splitting, in other words, spectrally resolving transmitted light into different angles all belonging to a single angular half-space with respect to the normal. This functionality results upon illumination of the un-patterned side of the structure with white light, i.e., “reverse illumination”, leading to emergence of a discrete set of colour-sorted beams from the patterned side, each traveling along a different, pre-defined angle to one side of the normal only (Fig. 3, top panel). For experimental characterization of this effect, the fabricated device was illuminated at normal incidence on its groove-free side using a TM-polarized supercontinuum white-light laser light with *H*-field parallel to the slit-length (oriented along the *y*-direction at *x* = 0, Fig. 3). The colour and intensity distribution of the transmitted light in a far-field plane located at a distance Δ*z* = 17.5 μm from the device exit surface was imaged using an inverted optical microscope (×100, NA = 0.75 microscope objective) and a colour-CCD camera (where the *x*-position of the transmitted light field relative to the center of the slit is calibrated by imaging the exit surface of a reference single-slit illuminated under identical conditions). By directly measuring the distance of the local intensity maximum of the red, green, and blue streaks relative to the center of the slit, Δ*x*, the diffraction angles for the red, blue, and green light are determined to be 0° ± 0.49°, 9.72° ± 0.47°, and 18.92° ± 0.44° respectively. The uncertainty in measurement of angles is one standard deviation, and calculated from the uncertainty in measuring the distances of the red, green, and blue streaks relative to the normal to the slit due to the finite pixel-spacing of the CCD camera. The measured angles are close to the angles specified for angle-selective colour-filter operation under “forward illumination” (0°, 10°, and 20° respectively), verifying the time-reversal symmetric behavior expected for any linear device. Note that the same spectrally resolved angular output response can be achieved for any angle of “reverse illumination” on the un-patterned side, the slit acting as a spatial filter. This approach to map the wavelength of incident radiation to a given angle can also be readily extended to more than three input wavelengths (e.g., five, by applying reciprocity to the result of Supplementary Fig. 7) allowing hyperspectral imaging where a spectral image cube can be directly acquired in a single exposure using a two-dimensional array of such devices coupled to an imaging chip. In contrast to other spectral imaging techniques, the colour-sorting approach presented here does not rely on filters or scanning interferometers that require long acquisition times for spectral-cube measurements. The multi-colour functionality achieved here with a single-device stands in contrast to the mono-colour functionality, characteristic of periodic plasmonic structures that require physically separate structures to achieve a full set of discrete colour responses^16,17,46,53,54^.

### Bullseye directional light filter

**Fig. 4:**
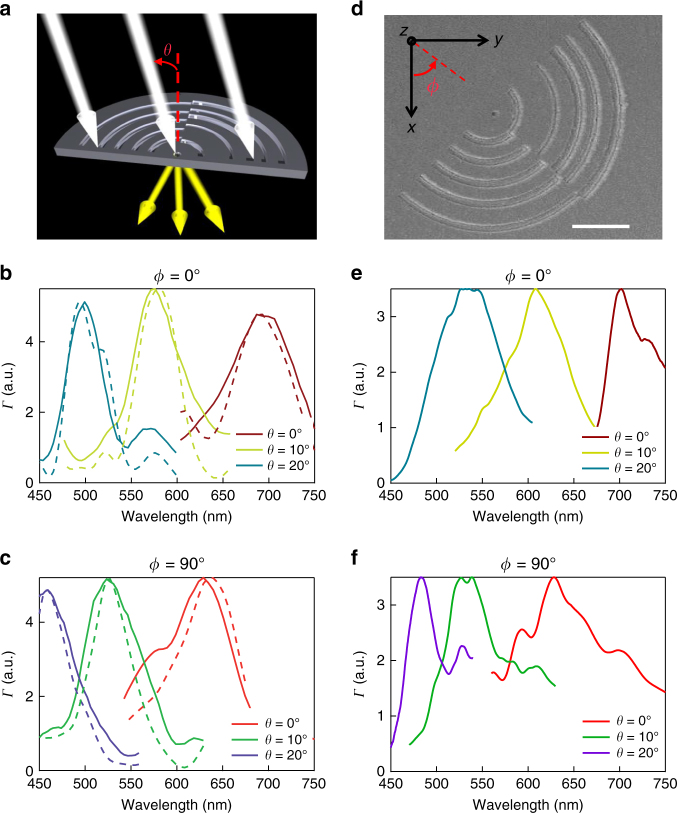
Aperiodic bullseye directional light-filter. **a** Schematic illustration of the light transmission through an aperiodic bullseye structure consisting of two distinct radially integrated linear aperiodic slit-groove structures. **b**, **c** Relative transmission (*Γ*) spectra calculated using the interference model of Eq. (3) (solid lines) and FDTD simulations (dashed lines) for *θ* = 0°, 10°, and 20° at both *ϕ* = 0° (**b**) and 90° (**c**). The calculated and simulated spectra agree with each other and demonstrate the incident angle (*θ*) and sample orientation (*ϕ*) dependent multi-spectral response of the device. **d** Top-down scanning-electron-microscope image of the bullseye device fabricated following the same procedure as the linear structure. The central circular aperture diameter in the bullseye structure is 150 nm and the scale bar represents 4 μm. The sample orientation (*ϕ*) relative to the axis of the bullseye is defined in the inset. **e**, **f** Experimentally measured *Γ* spectra corresponding to simulated spectra in **b**, **c**, respectively

Angle-selective colour filtering can also be realized by replacing an input surface decorated with an aperiodic collection of linear grooves surrounding a linear subwavelength-width slit, with one decorated with an aperiodic collection of circular concentric grooves surrounding a circular subwavelength-diameter aperture, forming an aperiodic bullseye pattern (Fig. 4a). Such a structure offers an additional rotational degree of freedom in obtaining different output spectra under white-light illumination for a given collection of input angles for a fixed plane of incidence and fixed TM polarization with respect to the surface (*H*-field vector in the plane of the bullseye). This is achieved by dividing up the bullseye into a discrete number of angular sectors (Fig. 4a, showing two such sectors), each having a distinct aperiodic groove arrangement. Each angular sector can then be individually addressed for a unique target colour response as a function of polar angle, *θ*, with respect to the principle axis of the aperture, by rotating the bullseye about that axis to an azimuthal angle, *ϕ*, such that the direction of the *H*-field vector is azimuthally centered within that sector. To implement such a *ϕ* addressable, azimuthal angle-selective colour-filter, a bullseye with two different functional sectors was designed using the optimization algorithm incorporating the one-dimensional interference model of Eq. (3), treating the curved grooves in a manner equivalent to the linear grooves. The device targets three specific illumination angles for colour sorting into two staggered sets of output wavelengths in the visible (listed in Supplementary Table 3) for *ϕ* = 0° and 90°, respectively. The model-calculated transmission spectra relative to that of an isolated circular aperture, *Γ*, for a bullseye structure consisting of two distinct aperiodic angular sectors, arranged orthogonally at *ϕ* = 0°and 90°, and probed at three different polar angles of incidence (*θ* = 0°, 10°, and 20°) show a distinct spectral peak, one for each of the six unique illumination conditions (Fig. 4b, c, solid lines), closely matching the target values (Supplementary Table 3). The corresponding relative transmission spectra numerically simulated using the FDTD technique (Fig. 4b, c, dashed lines), where Ag-film thickness *h* = 250 nm and aperture-diameter *d*_0_ = 150 nm are assumed, show remarkable agreement with the model generated spectra. The aperiodic bullseye was experimentally implemented (Fig. 4d) on an ITO coated fused-silica substrate using the same fabrication sequence as the linear slit-groove device (Methods section, and Supplementary Fig. 6). A reference device consisting of an isolated aperture of identical dimensions through the same Ag-film was also fabricated by FIB milling. The spectral transmission characteristics of the device, at its two azimuth orientations (*ϕ* = 0°and 90°), was measured by illuminating it with a TM-polarized supercontinuum white light laser (*H*-field vector in the plane of the bullseye) at three angles of incidence (*θ* = 0°, 10°, and 20°) for a fixed plane of incidence. The experimentally measured relative transmission spectra at each angle of incidence (normalized to that of the isolated reference aperture) are shown in Fig. 4e, f. A close match to the model predictions are obtained: namely, six distinct spectral peaks at each of the illumination conditions, and peak positions closely matching both target and analytic model-computed values (Supplementary Table 3). The multi-spectral response of the aperiodic bullseye structure wherein a particular spectrum is directly related to a specific directionality of the incident beam (*θ*) and rotational orientation of the device (*ϕ*) suggests applications as a directional light sensor in three-dimensions.

### Narrow linewidth refractive index sensing

**Fig. 5:**
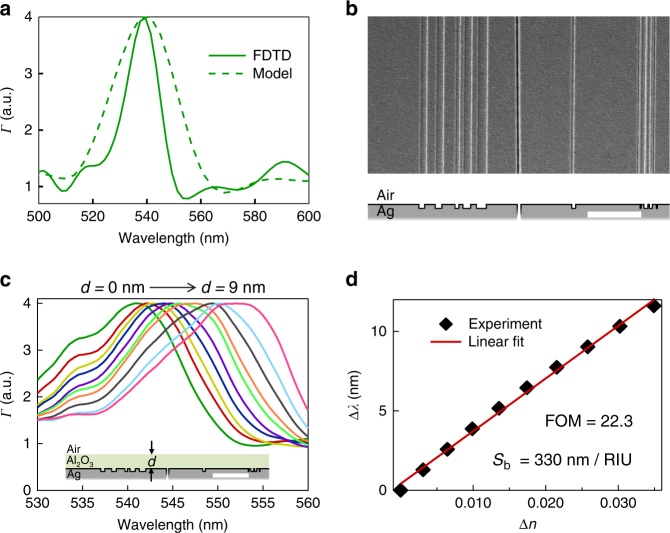
Linewidth optimization and refractive index sensing. **a** Spectral response, calculated using the interference model of Eq. (3) (dashed lines) and full-field FDTD simulations (solid lines), of a linewidth optimized aperiodic slit-groove device using Ag as the substrate upon illumination with a TM polarized light at *θ* = 0°. The relative transmission (*Γ*) exhibits a peak at 540 nm with a linewidth of 14.1 nm corresponding to a resonance quality factor of ≈38. **b** Top-down SEM image of the optimized device fabricated using the procedure outlined in Supplementary Fig. 6 along with the model predicted surface cross-section profile of the optimized device at a Ag-air interface. Scale bar represents 2 μm. **c** Experimentally measured spectral response as a function of increasing Al_2_O_3_ layer thickness, varying from 0 to 9 nm, for the device in **b**. Inset shows the surface cross-section profile of the optimized device with top Al_2_O_3_ layer. Scale bar represents 2 µm. **d** Peak spectral shift (Δ*λ*) vs. refractive index change (Δ*n*) for the data in **c**, resulting in a bulk sensitivity *S*_b_ = 330 nm/RIU and a figure-of-merit (FOM) ≈ 22.3. The FOM value achieved here is comparable to that of the state-of-the-art surface plasmon resonance sensors^56^

The aperiodic colour filters designed in this study are limited to five grooves each on both sides of the central slit within a lateral device footprint of ~10 µm, and the structural parameters of each groove was optimized to achieve angular colour selectivity at multiple angles of incidence simultaneously. However, for alternate applications such as refractive-index sensing, it is straightforward to redefine the angular and spectral target constraints input to the optimization algorithm to instead perform linewidth optimization (at the cost of angular selectivity) for a single angle of incidence within the same device footprint. Spectral linewidth is inversely related to the figure-of-merit (FOM), which is a metric used to compare the performance of refractive index optical sensors, and is defined as: FOM = ${S_{\rm{b}}}{\rm{/\Delta }}{\lambda _{1/2}}$, where ${S_{\rm{b}}} = {\rm{\Delta }}\lambda {\rm{/\Delta }}n$ is the bulk index-sensitivity of the device, Δ*λ* is the spectral peak shift for a change Δ*n* of the refractive index of the surrounding media, and Δ*λ*_1/2_ is the full-width at half-maximum linewidth of the spectral peak^55^. Here, we experimentally implement an aperiodic linear slit-groove Ag structure for which white-light illumination of the groove-decorated side at normal incidence yields narrow linewidth transmission at a center wavelength of 540 nm. The optimization algorithm yields an aperiodic device configuration for which the modeled transmission spectrum under illumination at *θ* = 0° exhibits a distinct peak at a center wavelength of 540 nm characterized by narrow linewidth Δ*λ*_1/2_ ≈ 14.1 nm (Fig. 5a, dashed line). The corresponding relative transmission spectra numerically simulated using the FDTD technique (Fig. 5a, solid line), where Ag-film thickness *h* = 250 nm and slit-width *W* = 100 nm are assumed, show remarkable agreement with the model generated spectra. The aperiodic sensing device was experimentally implemented (Fig. 5b) on an ITO coated fused-silica substrate using the same fabrication sequence as the other aperiodic devices (Methods section, and Supplementary Fig. 6). To evaluate the performance of a refractive-index sensor based on the aperiodic slit-groove array, we expose the Ag-air interface to a superficial perturbation in index of refraction under the form of ultra-thin Al_2_O_3_ layers of index *n* = 1.77 of thickness ranging from 1 to 9 nm (Fig. 5c), conformally deposited using atomic layer deposition. Nanometer-scale spectral shifts of the spectral peak to longer wavelengths, as a function of increasing layer thickness (Fig. 5c, d), are easily resolvable due to the narrow resonance linewidth characteristic of the device. The experiments yield a refractive index wavelength sensitivity ${S_{\rm{b}}} = {\rm{\Delta }}\lambda {\rm{/\Delta }}n$ ≈ 330 nm·RIU^−1^, along with an FOM = ${S_{\rm{b}}}{\rm{/\Delta }}{\lambda _{1/2}}$ ≈ 22.3 that is comparable to that of a state-of-the-art, commercial surface-plasmon resonance (SPR) sensor based on Kretschmann configuration excitation^56^ as well as plasmonic interferometric sensors^57^. The effective refractive index change (Δ*n*) is determined using an effective medium approximation of dielectric bi-layer coating the metal surface into a single dielectric medium of refractive index *n*_eff_ (Supplementary Note 2). Finally, the applicability of multi-band spectral transmission of the aperiodic devices for multiplexed plasmon sensing applications is demonstrated for five spectral peaks spanning the visible frequency range (Supplementary Note 3).

## Discussion

The performance (in terms of spectral or spatial crosstalk, and sensing FOM) of the aperiodic devices studied here is primarily limited by the losses in the as-deposited evaporated Ag-film, wherein the 1/e SPP decay-length (*L*_SPP_) placed an upper-limit on the lateral footprint of the device to *L* ≤ 10 µm. The experimentally measured value of *L*_SPP_ for the evaporated Ag-films used in the experiments, at a FS wavelength of 690 nm, is determined to be *L*_SPP_ = 7 µm (Supplementary Note 4). This limit on lateral device footprint was also determined from the dependence of spectral response of the aperiodic colour filter on the number of grooves wherein the spectral response saturated with increasing number of grooves (reaching saturation at *N* = *M* = 5, Supplementary Fig. 13). However, based on recent progress in using the template-stripping approach to create ultra-smooth Ag films with typical values of *L*_SPP_ ranging from 30 to 80 µm^58,59^, utilizing template-stripped Ag-films would be one very straightforward approach to enhance the performance of these devices. The template-stripping approach also directly lends itself towards fabricating the inverse groove structures onto reusable Si templates where groove-depth along with its width and location can be used as a free-parameter to further improve the flexibility in device-design. Finally, regarding the interference model, incorporating higher-order SPP–SPP and SPP-incident light interactions would allow for a more accurate prediction of the resonance lineshape and spectral peaks that closely match those predicted by numerical solvers or measured experimentally.

In conclusion, we have developed a robust interference-based first-order analytical model to calculate the transmission properties of plasmonic devices with aperiodic topologies. Incorporating the model into a structural optimization algorithm enables straightforward design of ultra-compact directional light-filters and colour-sorters exhibiting angle- or spectral-tunable optical responses with both high contrast and low spectral or spatial crosstalk, hinting at promising applications in solar-energy harvesting, optical signal multiplexing, and high-FOM refractive index sensing. By substituting, as the core of the optimization process, an analytical physical model for brute-force numerical simulation, we demonstrate a simple and efficient route towards leveraging aperiodic topologies to achieve devices with flexible and multi-spectral optical functions that are fundamentally not achievable using periodic architectures.

## Methods

### Fabrication

The aperiodic colour-filter structures are fabricated on 20-nm-thick ITO coated fused silica substrates. Electron-beam lithography at 100 keV was used to expose the inverse groove pattern on the 100-nm-thick poly-methyl methacrylate (PMMA) resist spun-coated on the substrates. After the exposure, PMMA was developed for 60 s in methyl isobutyl ketone followed by a 30 s rinse in isopropyl alcohol. Electron-beam evaporation was used to deposit a 5-nm-thick Cr adhesion layer, followed by a 100-nm-thick Ag-film. A 12-hour soak in acetone was used for lift-off leaving rectangular islands of Ag at the location of the exposed areas. A second Ag deposition of thickness 150 nm was performed using electron-beam evaporation in order to elevate the groove pattern by an optically thick layer above the plane of the substrate. Finally, FIB milling was used to create a 100-nm-wide, 10-μm-long central through slits (or 150-nm-diameter circular through apertures). The fabrication steps are schematically outlined in Supplementary Fig. 6. For the sensing device, the patterned Ag-air interface was conformally coated with an ultra-thin layer of Al_2_O_3_ of thickness ranging from 1 to 9 nm using atomic layer deposition.

### Measurements

For experimental characterization, the samples were illuminated using a TM polarized supercontinuum white light laser (emission wavelength range: 400–2000 nm) at various angles of incidence, and sample orientation. The angle of incidence at the sample was controlled using a motorized rotation-tilt-mirror mounted on a linear translation stage. For spectral measurements, the light transmitted through the devices was collected using ×100 microscope objective (NA = 0.75) and directed to a grating spectrometer connected to a cooled Si-CCD camera. In each case, the transmitted intensity from the linear slit-groove (bullseye) device was normalized to that of an isolated reference slit (circular aperture). For spectral splitting experiments, accurate referencing of the focal plane of the optical microscope relative to the exit surface of the device was achieved by imaging the exit surface (Δ*z* = 0 μm) of the device. The colour and intensity distribution of the transmitted light in a far-field plane located at a distance Δ*z* = 17.5 μm from the device exit surface was imaged using an inverted optical microscope (×100, NA = 0.75 microscope objective) and a colour-CCD camera. The *x*-position of the transmitted light field relative to the center of the slit was calibrated by imaging the exit surface of a reference single-slit illuminated under identical conditions. By directly measuring the distance of the local intensity maximum of the red, green, and blue streaks relative to the center of the slit, Δ*x*, the diffraction angles, $\theta = {\rm{ta}}{{\rm{n}}^{ - 1}}\left( {{\rm{\Delta }}x{\rm{/\Delta }}z} \right)$ for the red, blue, and green light were determined.

### Data availability

All data needed to evaluate the conclusions in the paper are present in the paper and the Supplementary Information. Additional data available from the corresponding author upon reasonable request.

## Electronic supplementary material

Supplementary Information
