# Supplementary material for: Aperiodic nanoplasmonic devices for directional colour filtering and sensing
Source: Nat Commun. 2017 Nov 7;8:1347. doi: 10.1038/s41467-017-01268-y (PMC5676867; doi:10.1038/s41467-017-01268-y)
Supplement: Supplementary file 1 — Supplementary Information [file 41467_2017_1268_MOESM1_ESM.pdf]

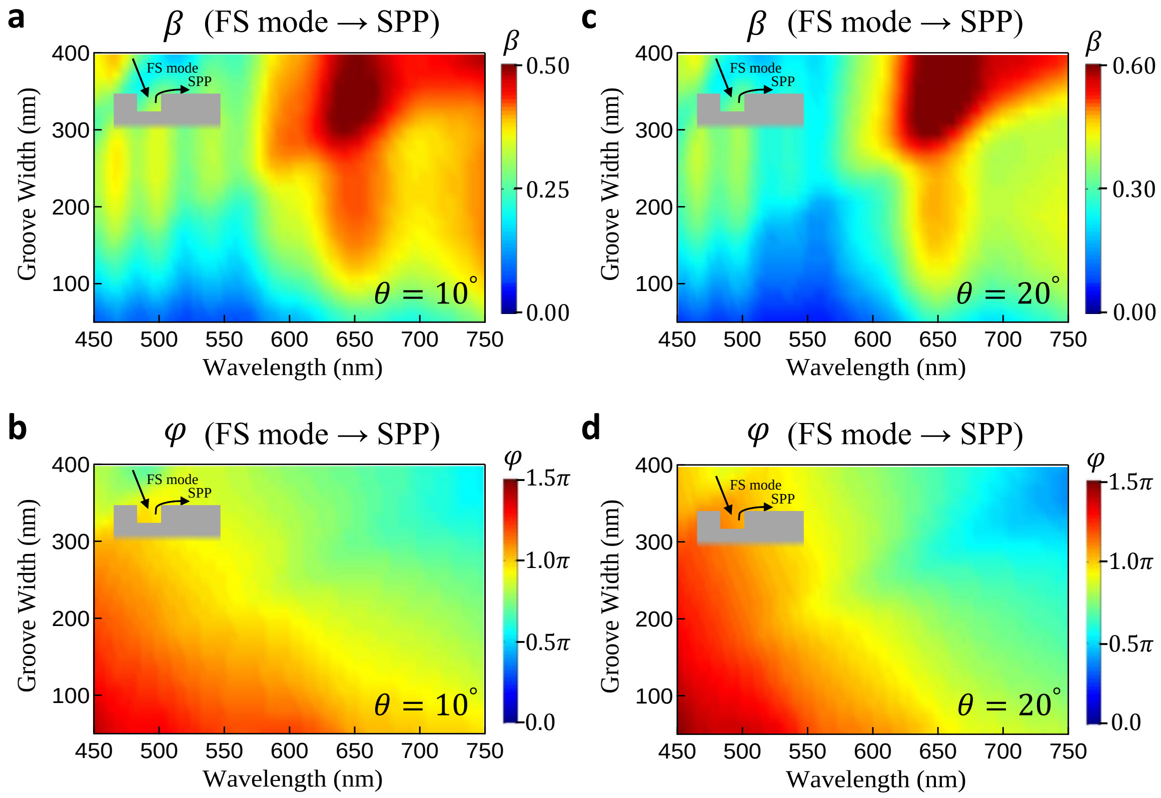

Supplementary Figure 1 | Variation in coupling coefficient β and phase shift φ with θ. Variation in β and φ as a function of both groove-width (w: 50 nm to 400 nm) and free-space wavelength (λ_0: 450 nm to 750 nm), for illumination at $\theta = 10°$ (**a** and **b,** respectively) and $\theta = 20°$ (**c** and **d,** respectively), and fixed groove-depth ($t = 100$ nm) at a Ag-air interface. The variation in β, φ, β' and φ' at $\theta = 0°$ is summarized in Figure 1 of the manuscript and, for consistency, it was verified that β' and φ' does not vary with θ.

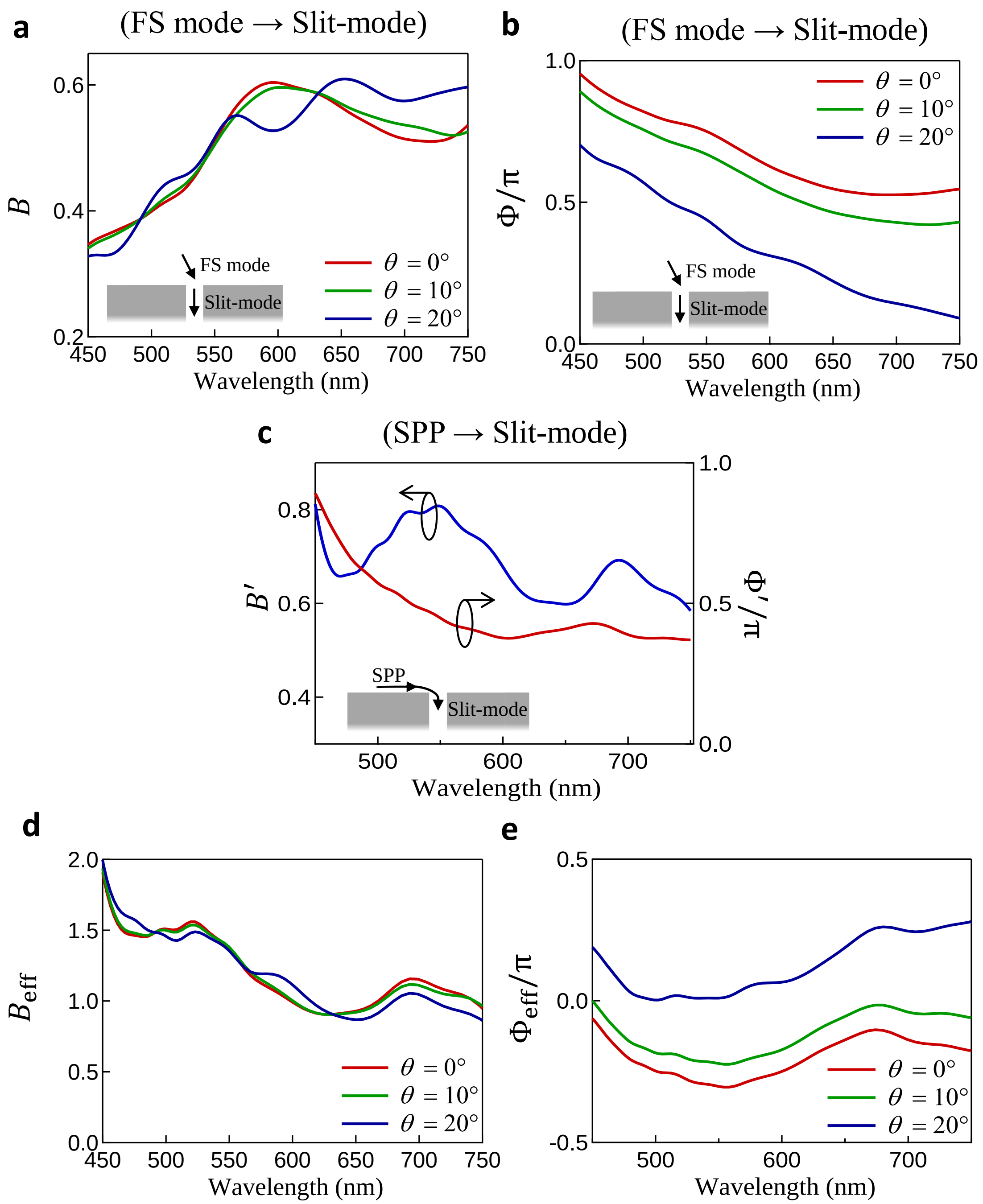

Supplementary Figure 2 | Coupling of free-space mode and SPP into a slit-mode. Variation in **a,** amplitude coupling coefficient $B(W, \theta, \lambda_0)$ and **b,** phase shift $\Phi(W, \theta, \lambda_0)$ for a plane wave coupling into guided modes inside a slit of width W = 100 nm at a Ag-air interface for λ_0 = 450 nm to 750 nm at illumination angles of θ = 0°, 10° and 20°. Variations in **c,** coupling coefficient $B'(W, \lambda_0)$ and phase-shift $\Phi'(W, \lambda_0)$ for SPP modes coupling into a guided mode inside a slit of the same width. Corresponding variations in the calculated values of **d,** B_{eff} and **e,** Φ_{eff} under identical illumination conditions.

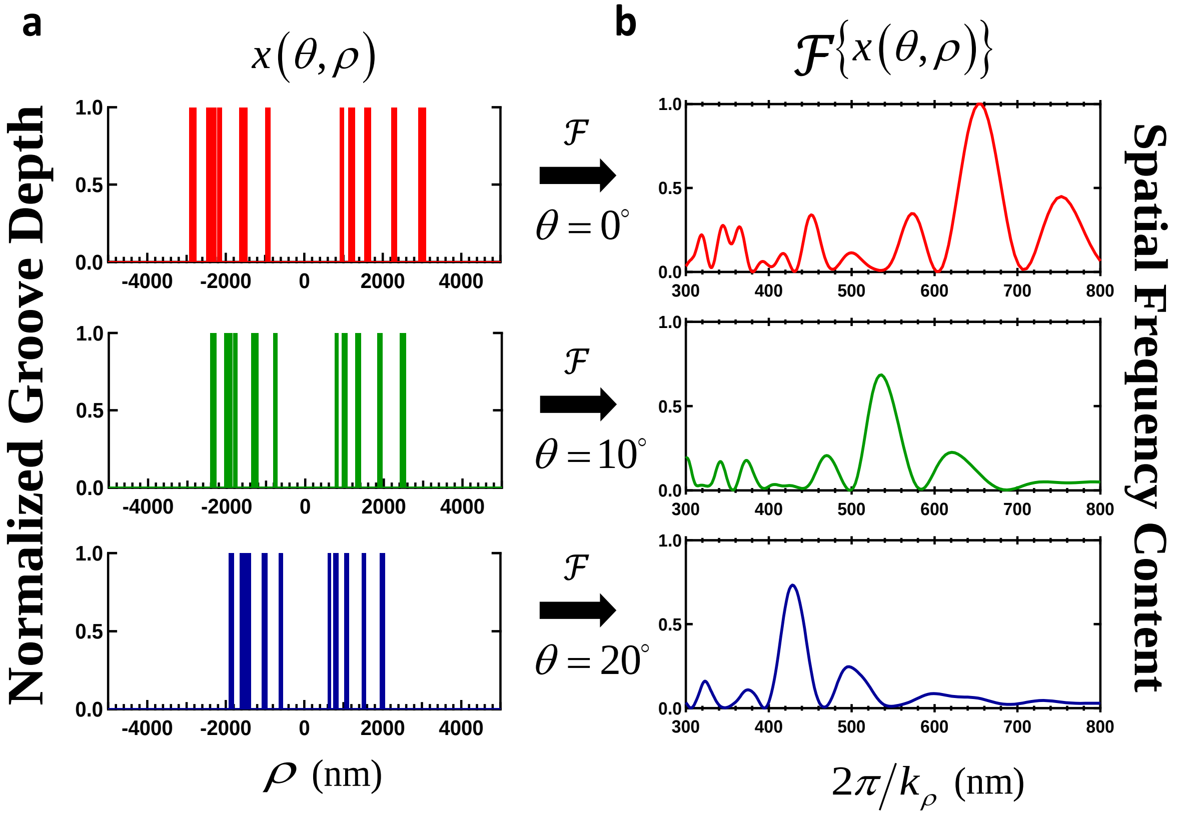

Supplementary Figure 3 | Spatial-frequency content of the aperiodic colour-filtering device. **a,** The real-space waveform representing the groove locations ρ, and their widths w, as projected onto the plane of incidence at each of the three angles of incidence (θ = 0°, 10° and 20°), for the aperiodic colour-filter device described in Figure 2 of the manuscript. As the angle of incidence θ increases, the perceived groove location and width of the aperiodic groove array varies as $\rho_{\text{eff}} = \rho(1 - \sin\theta)$ and $w_{\text{eff}} = w(1 - \sin\theta)$ respectively. **b,** Spatial Fourier-transform of the real-space groove-waveform depicting the associated reciprocal wave-vectors in inverse k-space. As expected, the aperiodic device exhibits dominant spatial-frequency content at wavelengths that agree with the modeled and experimentally measured spectral outputs (Figure 2c and 2e of the manuscript, respectively).

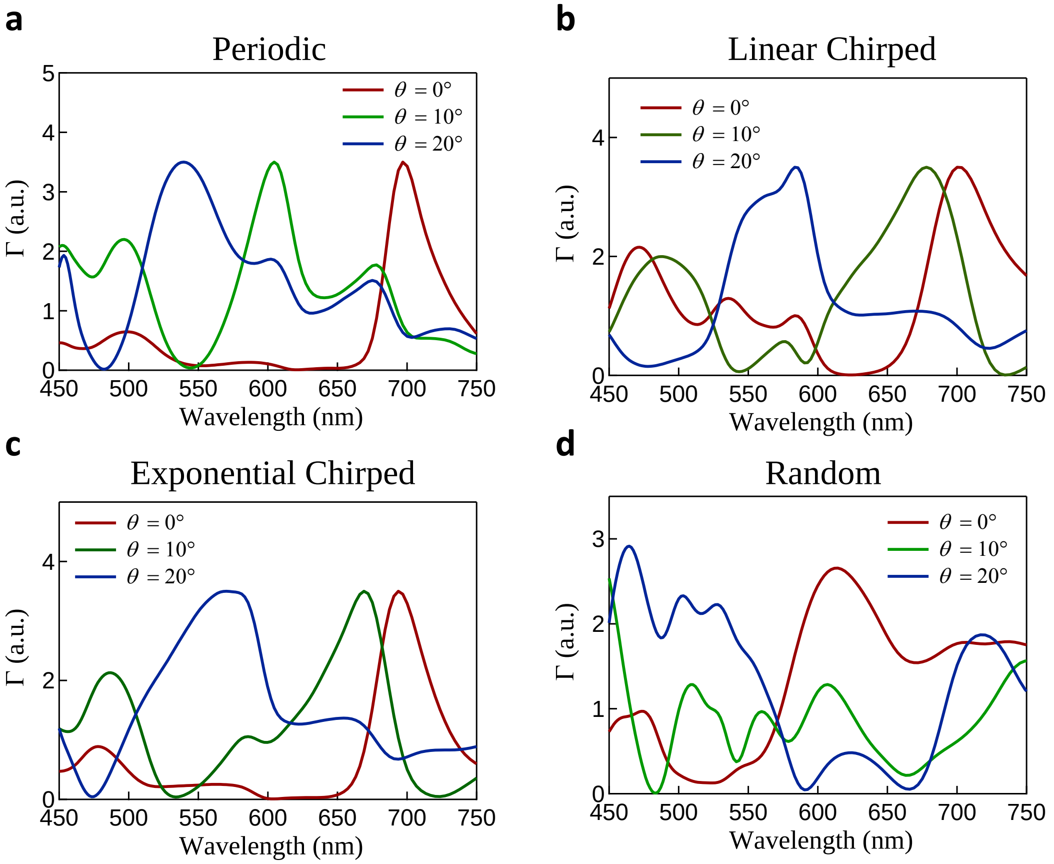

Supplementary Figure 4 | Incident-angle dependent transmission spectra for periodic, chirped and random slit-groove devices. FDTD calculated relative transmission spectra (Γ), at incident angles $\theta = 0°$, $10°$ and $20°$, for slit-groove devices where the grooves are arranged: **a,** periodically with pitch p = 630 nm; **b,** in a linear chirped geometry; and **c,** in an exponential chirped geometry around a central subwavelength slit. For the linear chirped device, the distance of each individual groove from the slit, x_n, follows the equation: $x_n = x_{n-1} + 2\pi/k_x$ where $n > 0$, $k_x = 2\pi/x_0 + 0.0005n$ and $x_0 =$ 630 nm. For the exponentially chirped device: $x_n = x_{n-1} + 2\pi/k_x$ where $n > 0$, $k_x = (1.01)^n\ (2\pi/x_0)$ and $x_0 = 620$ nm. **d,** Relative transmission spectra (Γ), at incident angles $\theta = 0°$, $10°$ and $20°$, for a device with random placement of ten grooves within the 10 μm-wide lateral footprint of the device. For each simulated device, Ag-film thickness $h = 250$ nm, slit-width $W = 100$ nm, groove width $w = 50$ nm and groove depth $t =$ 50 nm was used.

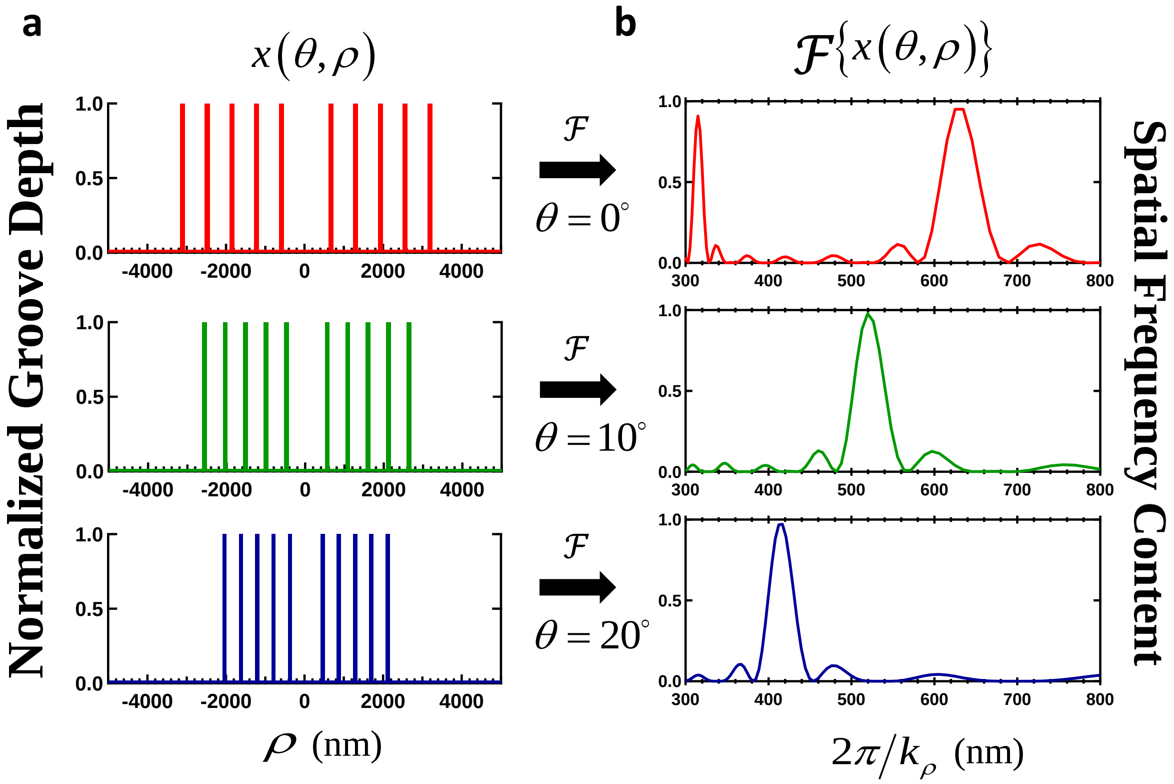

Supplementary Figure 5 | Spatial-frequency content of an equivalent periodic groove device. **a,** The real-space waveform representing the groove locations ρ, and their widths w, as projected onto the plane of incidence at each of the three angles of incidence (θ = 0°, 10° and 20°) for a periodic groove device of periodicity p = 630 nm and constant groove width W = 50 nm. As the angle of incidence θ increases, the perceived groove location and width of the periodic groove array device also varies as $\rho_{\text{eff}} = \rho(1 - \sin\theta)$ and $w_{\text{eff}} = w(1 - \sin\theta)$ respectively. **b,** Spatial Fourier-transform of the real-space groove-waveform depicting the associated reciprocal wave-vectors in inverse ***k***-space.

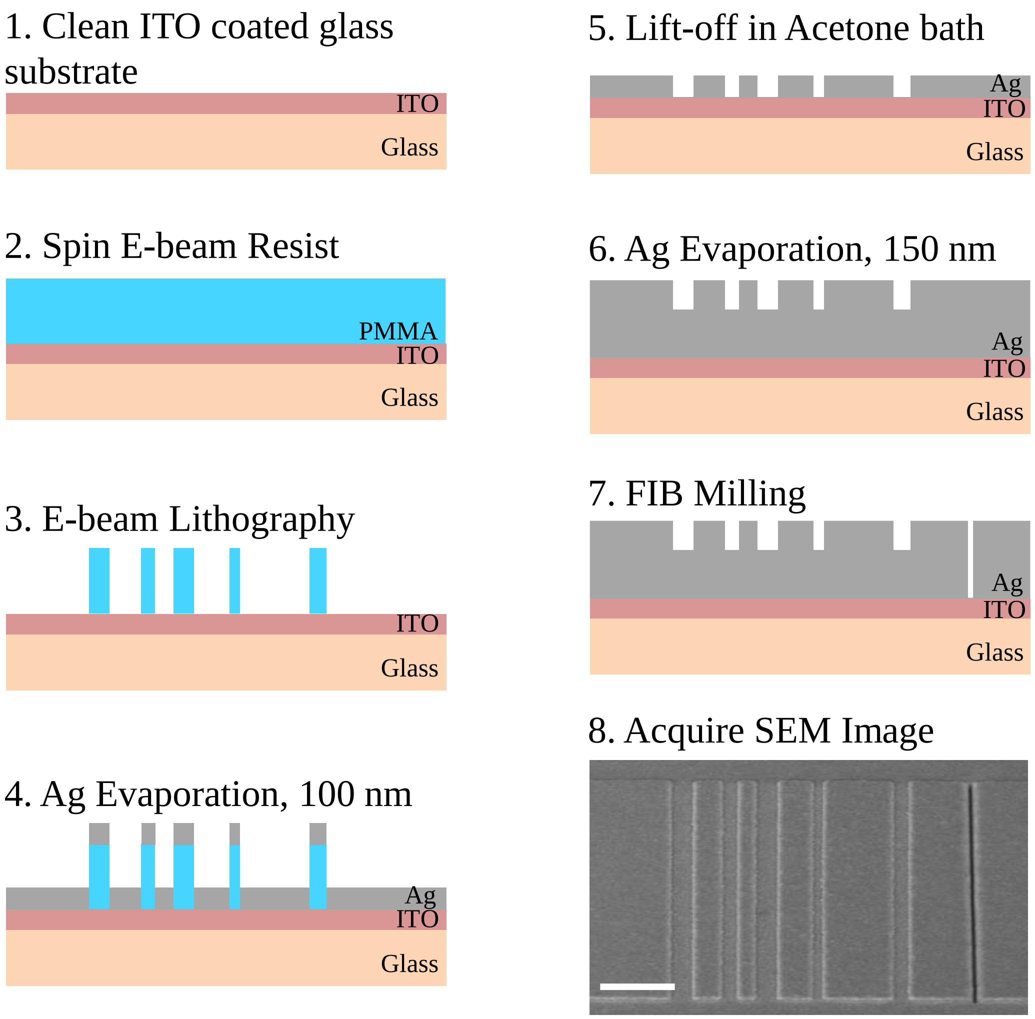

Supplementary Figure 6 | Nanofabrication process steps. E-beam resist (PMMA) was spin-coated on a pre-cleaned 20 nm thick ITO-coated fused silica substrate. E-beam lithography (at 100 keV) was used to expose the inverse groove pattern on the resist, and the exposed resist was subsequently developed for 60 s in MIBK followed by 30 s rinse in IPA. Using E-beam evaporation, a 5 nm thick Cr adhesion layer, followed by 100 nm thick Ag was deposited. Following deposition, lift-off was carried out by soaking the sample in Acetone for twelve-hours. The lift-off procedure leaves Ag islands at the location of the exposed regions. A second Ag deposition of thickness 150 nm was performed using electron-beam evaporation in order to elevate the groove pattern by an optically thick layer above the plane of the substrate. Finally, focused-ion-beam milling was used to create a 100 nm-wide, 10 µm-long central through slits (or 150 nm-diameter circular through apertures). The scale bar in the SEM image represents 2 µm.

Supplementary Note 1 | Plasmonic colour pixel analysis:

The optical contrast I_{C} of the aperiodic slit-groove device summarized in Figure 2 of the manuscript for the three spectral peaks with FWHM linewidths ($\Delta\lambda_{1/2}$) is calculated as:

$$I_{\mathrm{C}} = \frac{I_{\mathrm{ON}} - I_{\mathrm{OFF}}}{I_{\mathrm{ON}} + I_{\mathrm{OFF}}}, \quad (1)$$

where I_{ON} represents the spectral amplitude at the targeted wavelength of interest at corresponding incident angle (for *e.g.*, 690 nm at 0°) and I_{OFF} is the residual spectral amplitude at that same wavelength (690 nm) for other incident angles (10° and 20°). The device exhibits an optical contrast of up to 93 % and linewidths as narrow as 60 nm (Supplementary Table 1).

The angle-resolved spectral colour filtering property of the aperiodic plasmonic device has potential for applications as RGB colour pixels. In recent years, several periodic plasmonic colour-pixel designs that include array of apertures, slits or slit-grooves have been proposed for high-quality CMOS digital image sensor applications [1-4]. Here, we quantitatively measure the spectral crosstalk, or bleed, which is a measure of the performance of a colour-filter, for the aperiodic angle-resolved colour-filters fabricated in this study (Figure 2 of the manuscript). Spectral crosstalk is a quantitative measurement of the overlap between various spectra in a device with a multi-band spectral response, and is defined as [4, 5]

$$C_i/C_j = \left\{ \int_{\Delta\lambda_i} \Gamma(\theta_j, \lambda_j)\, d\lambda \Big/ \int_{\Delta\lambda_i} \Gamma(\theta_i, \lambda_i)\, d\lambda \right\}_{i \neq j}, \quad (2)$$

where $\Delta\lambda_i$ is the integration range extending over the linewidth $\Delta\lambda_{1/2}$ for a relative spectral transmission $\Gamma(\theta, \lambda)$ peak at λ_i. Each integrated spectral range is represented by $C_{i/j}$ with *i* and *j* =1, 2 or 3 for the three-peaks, respectively and $i \neq j$. The ideal spectral crosstalk for a colour-pixel, given by equation (2), is 0 % – indicating that there is no spectral overlap between neighboring spectral peaks. The aperiodic plasmonic device studied here is able to achieve spectral crosstalk values that are comparable to conventional colour filters (Supplementary Table 2). Note here that the performance specifications of the experimentally implemented aperiodic colour-filter structuresincluding spectral linewidth, optical contrast and spectral

crosstalk, are all comparable to state-of-the-art plasmonic counterparts that rely on periodic nanostructures [6-8]. The optimization algorithm incorporating the interference model allows us to achieve angle resolved full-colour response from a single-pixel device on a micron-scale device footprint.

	$\Delta\lambda_{1/2}$, FWHM	I_C (690 nm, 0°)	I_C (545 nm, 10°)	I_C (480 nm, 20°)
(690 nm, 0°)	60 (120) nm	N/A	60 (87) %	93 (70) %
(545 nm, 10°)	60 (85) nm	80 (93) %	N/A	84 (95) %
(480 nm, 20°)	38 (55) nm	74 (72) %	83 (88) %	N/A

Supplementary Table 1 | Linewidth and optical contrast of the aperiodic color filter. Experimentally measured spectral peak characteristics (linewidth $\Delta\lambda_{1/2}$ and optical contrast I_C) for the aperiodic colour filter device at θ = 0°, 10° and 20° corresponding to spectral peaks at λ = 690 nm, 545 nm and 480 nm. The values predicted by FDTD-calculations are shown in parenthesis for reference.

	Red (690 nm)	Green (545 nm)	Blue (480 nm)
Red (690 nm)	N/A	10 (5)	15 (6)
Green (545 nm)	15 (9)	N/A	14 (32)
Blue (480 nm)	29 (0)	16 (17)	N/A

Supplementary Table 2 | Spectral crosstalk exhibited by the aperiodic color filter. Experimentally measured spectral crosstalk exhibited by the aperiodic colour filter along with the crosstalk values for conventional filters shown in parentheses [2].

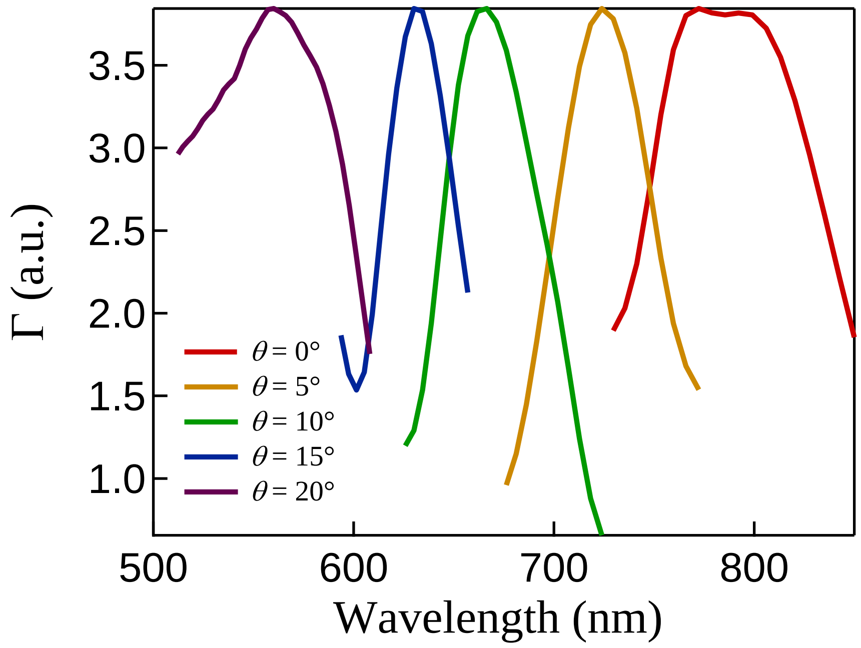

Supplementary Figure 7 | Multispectral Plasmonic Response. An aperiodic device designed on Au to exhibit five angle-dependent spectral peaks spanning the visible-near infrared spectral range. The simultaneous multi-band spectral response from a micron-scale single-pixel size device suggests application for spectral colour-sorters in areas such as multiplexed sensing or hyperspectral imaging.

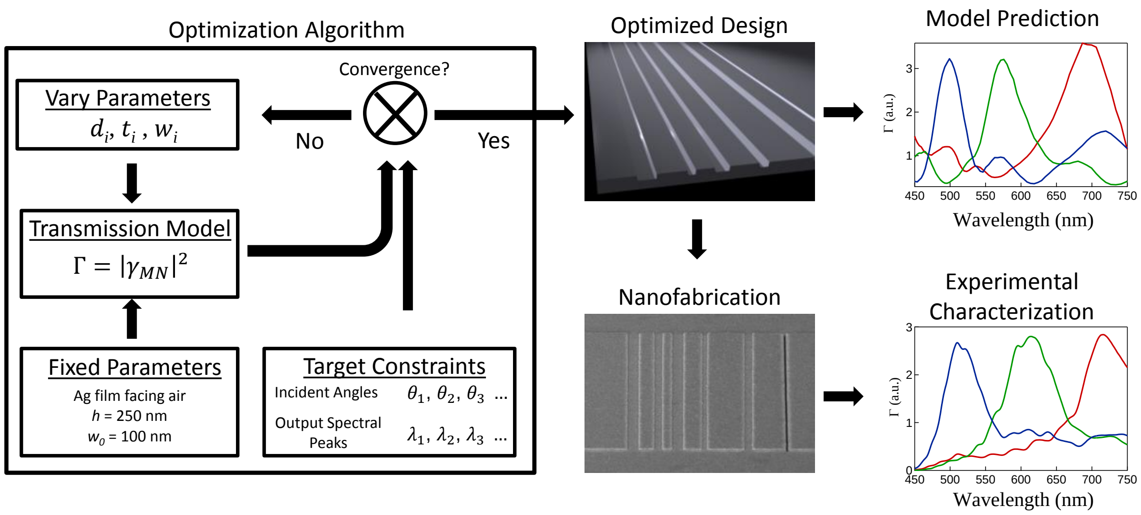

Supplementary Figure 8 | Design and implementation process summary. The complete process-flow for the design and optimization, nanofabrication and experimental characterization of an aperiodic plasmonic device with target performance specifications. The optimization process uses the transmission model described in the manuscript along with design and target constraints to optimize for the final aperiodic groove-design. The nanofabrication procedure (outlined in Supplementary Figure 6) is used to fabricate the device, and experimental characterization using a supercontinuum white-light laser as the illumination source and a spectrometer coupled to a cooled Si-CCD, is used to measure the relative spectral transmission intensity. Finally, model calculated relative transmission spectra for the optimized device design are compared with the target specification and experimentally measured spectra.

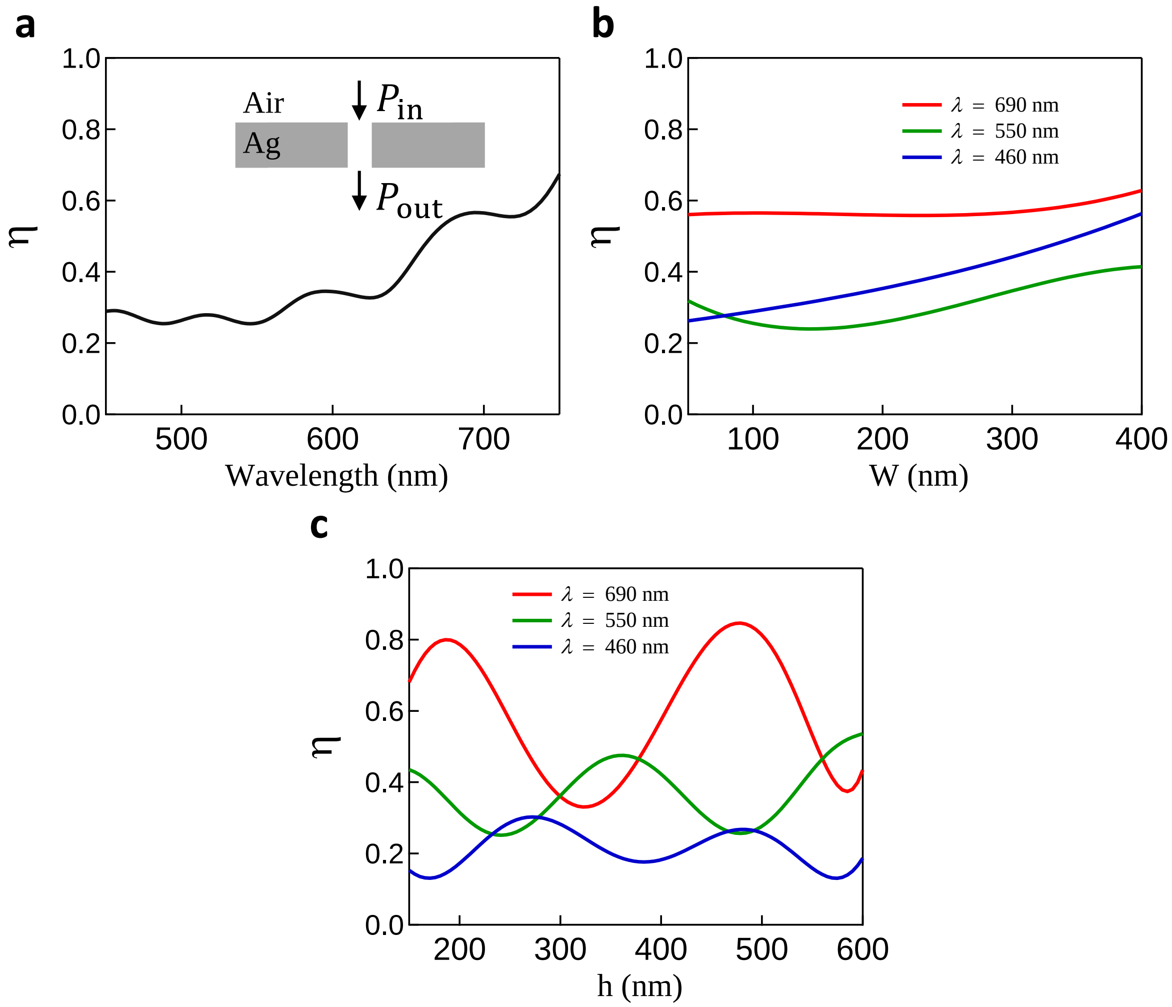

Supplementary Figure 9 | Absolute power transmission efficiency of a single-slit. a, FDTD calculated power transmission efficiency η of a single-slit of width W = 100 nm fabricated on a Ag substrate of thickness h = 250 nm. Power transmission efficiency here is defined as $\eta = P_{out}/P_{in}$ where P_{in} is the power of a normally incident TM-polarized wave on the slit and P_{out} is the power transmitted by the slit into the far-field. **b,** Variation in η as a function of W for h = 250 nm at three incident wavelengths (λ_0 = 690 nm, 550 nm and 460 nm). As expected, η increases with increasing slit-width W. **c,** Variation in η as a function of h for W = 100 nm at λ_0 = 690 nm, 550 nm and 460 nm where the oscillatory modulation in transmission *vs.* h is due to Fabry-Perot interference of the guided-mode propagating within the slit.

		$\theta = 0°$	$\theta = 10°$	$\theta = 20°$
$\phi = 0°$	Target	700 nm	600 nm	500 nm
	Model	695 nm	575 nm	495 nm
	Measured	702 nm	608 nm	535 nm
$\phi = 90°$	Target	640 nm	555 nm	460 nm
	Model	620 nm	520 nm	454 nm
	Measured	628 nm	532 nm	484 nm

Supplementary Table 3 | Designed, modeled and experimentally measured spectral peak positions. Targeted spectral-peak positions for the aperiodic bullseye structure (Figure 4 of the manuscript) under various illumination conditions, compared to the peak-positions from the interference-model for the optimized structure, and to those measured experimentally from the fabricated device.

Supplementary Note 2 | Effective index calculation of a bi-layer dielectric medium:

In order to accurately determine the figure-of-merit (FOM) of the aperiodic plasmonic sensor (studied in Figure 5 of the manuscript), an accurate determination of the effective change in refractive-index (Δn), when the metal (Ag, medium 1) surface is coated with nanometer-scale thick dielectric (Al_2O_3, medium 2) layer, is required. This is achieved by converting the bi-layer Al_2O_3/vacuum dielectric over-coating (medium 2 and 3 in Supplementary Figure 10a) into an effective bulk dielectric of refractive index n_{eff} (Supplementary Figure 10b).

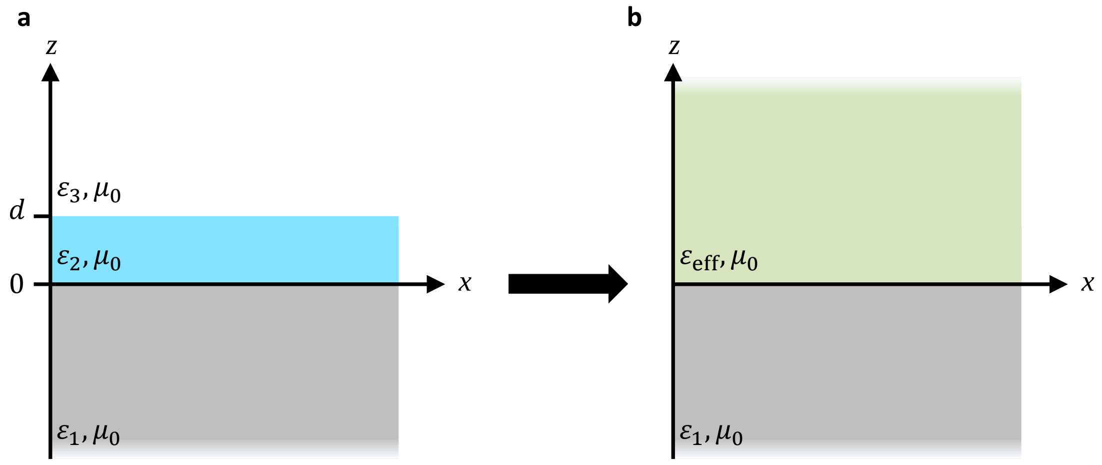

Supplementary Figure 10 | Effective medium approximation of dielectric bi-layer into a single effective dielectric medium. a, A 3-layer system. Medium 1 (described by complex dielectric permittivity ε_1 and free-space permeability μ_0) and medium 3 (ε_3, μ_0) are assumed to be of semi-infinite thickness on either side of medium 2 (ε_2, μ_0) of thickness d. All three mediums are assumed to be non-magnetic, linear, isotropic, and homogeneous. **b,** Mediums 2 and 3 together are approximated to be semi-infinite effective medium of complex dielectric permittivity ε_{eff} and free-space permeability μ_0.

The conversion requires the calculation of the lowest order bound modes in Supplementary Figure 10a. Assuming TM polarization, Ampere's law $\vec{\nabla}\times\vec{H} = -i\omega\varepsilon_0\varepsilon_j\vec{E}$ gives the full field expressions for each medium j (j =1, 2, 3). This is expressed in equations 3, 4, and 5 using Cartesian unit vectors $\hat{a}_x$, $\hat{a}_y$, and $\hat{a}_z$ where ω is the angular frequency, ε_j is the medium dielectric constant, and ε_0 the permittivity of free-space. The field amplitudes for each region are given by H_1 (region 1), $H_{2,1}, H_{2,2}$ (region 2), and H_3 (region 3). The wave-numbers for each region are given by $k_j = \sqrt{\varepsilon_j}k_0$ where $k_0 = \omega/c_0$ and c_0 is the speed of light in free-space. Each k_j obeys the relation $k_j^2 = k_{j,x}^2 + k_{j,z}^2$.

($z < 0$)

$$\vec{H}_1 = \hat{a}_y \mathrm{H}_1 e^{-ik_{1,z}z} e^{ik_{1,x}} \quad (3.a)$$

$$\vec{E}_1 = \frac{-H_1}{\omega\varepsilon_0\varepsilon_1}\left(\hat{a}_x k_{1,z} + \hat{a}_z k_x\right) e^{-ik_{1,z}z} e^{ik_{1,x}} \quad (3.b)$$

($0 < z < d$)

$$\vec{H}_2 = \hat{a}_y\left(\mathrm{H}_{2,1}e^{ik_{2,z}z} + \mathrm{H}_{2,2}e^{-ik_{2,z}z}\right)e^{ik_{2,x}} \tag{4.a}$$

$$\vec{E}_2 = \frac{1}{\omega\varepsilon_0\varepsilon_2}\left[\hat{a}_x k_{2,z}\left(-H_{2,1}e^{ik_{2,z}z} + H_{2,2}e^{-ik_{2,z}z}\right)e^{ik_{2,x}} - \hat{a}_z k_x\left(H_{2,1}e^{ik_{2,z}z} + H_{2,2}e^{-ik_{2,z}z}\right)e^{ik_{2,x}}\right] \tag{4.b}$$

($z > d$)

$$\vec{H}_3 = \hat{a}_y \mathrm{H}_3 e^{ik_{3,z}z}e^{ik_{3,x}} \tag{5.a}$$

$$\vec{E}_3 = \frac{H_3}{\omega\varepsilon_0\varepsilon_3}\left(-\hat{a}_x k_{3,z} + \hat{a}_z k_x\right)e^{ik_{3,z}z}e^{ik_{3,x}} \tag{5.b}$$

The boundary conditions for this system are given by:

$$\begin{aligned} H_y(z=0^-) &= H_y(z=0^+) \\ H_y(z=d^-) &= H_y(z=d^+) \\ E_x(z=0^-) &= E_x(z=0^+) \\ E_x(z=d^-) &= E_x(z=d^+) \end{aligned} \tag{6}$$

Applying these boundary conditions to equations (3), (4) and (5) gives $k_{1,x} = k_{2,x} = k_{3,x} = k_x$ and:

$$H_1 = H_{2,1} + H_{2,2} \tag{7a}$$

$$\frac{k_{1,z}}{\varepsilon_1}H_1 = \frac{k_{2,z}}{\varepsilon_2}\left(H_{2,1} - H_{2,2}\right) \tag{7b}$$

$$H_3 e^{ik_{3,z}d} = H_{2,1}e^{ik_{2,z}d} + H_{2,2}e^{-ik_{2,z}d} \tag{7c}$$

$$\frac{k_{3,z}}{\varepsilon_3}H_3 e^{ik_{3,z}d} = \frac{k_{2,z}}{\varepsilon_2}\left(-H_{2,1}e^{ik_{2,z}} + H_{2,2}e^{-ik_{2,z}}\right) \tag{7d}$$

Eliminating the four *H*-field amplitudes from equations (7a) to (7d) gives the dispersion relation:

$$e^{i2k_{2,z}d} = \frac{\left(\frac{k_{3,z}}{\varepsilon_3}+\frac{k_{2,z}}{\varepsilon_2}\right)\left(\frac{k_{1,z}}{\varepsilon_1}+\frac{k_{2,z}}{\varepsilon_2}\right)}{\left(\frac{k_{3,z}}{\varepsilon_3}-\frac{k_{2,z}}{\varepsilon_2}\right)\left(\frac{k_{1,z}}{\varepsilon_1}-\frac{k_{2,z}}{\varepsilon_2}\right)} \tag{8}$$

A similar dispersion relation is obtained in [9], however, in equation (8) no initial assumptions about k_z in the three-regions is made.

Supplementary Table 4 shows the solutions of equation (8) for various medium 2 thicknesses d, ranging from $d = 0$ nm to $d \rightarrow \infty$. For the aperiodic sensing device (in Figure 5 of the manuscript): medium 1 is Ag, medium 2 is Al_2O_3, and medium 3 is free-space. At the sensor operating wavelength of 540 nm, this corresponds to $\varepsilon_1 = -10.5760 + 0.8383i$, $\varepsilon_2 = 3.1364$, and $\varepsilon_3 = 1$, using published values of dielectric constant for Ag [10]. For $d = 0$ nm, medium 2 makes no contribution and the values of $k_{1,z}$ and $k_{3,z}$ that satisfy equation (8) are complex and represent a bound-mode (Supplementary Table 4). The calculated value for k_x (Supplementary Table 4) also agrees with the theoretical prediction for a bound SPP-mode in a two-layer metallo-dielectric system, $k_x/k_0 = \sqrt{\varepsilon_1\varepsilon_3/(\varepsilon_1 + \varepsilon_3)} = 1.0505$ [11]. In the limit $d \rightarrow \infty$, medium 3 makes no contribution, and the values of $k_{2,z}$ that satisfies equation (8) is large and imaginary. The calculated mode for k_x (Supplementary Table 4) in this case also agrees with the theoretical prediction $k_x/k_0 = \sqrt{\varepsilon_1\varepsilon_2/(\varepsilon_1 + \varepsilon_2)} = 2.1079$ [11]. For intermediate values of d, an effective medium dielectric constant can be calculated by setting $k_x/k_0 = \sqrt{\varepsilon_1\varepsilon_{\text{eff}}/(\varepsilon_1 + \varepsilon_{\text{eff}})}$. Recognizing that the refractive index of a non-magnetic, isotropic medium is given by $n_j = \sqrt{\varepsilon_j}$ gives $k_x/k_0 = n_1 n_{\text{eff}}/\sqrt{n_1^2 + n_{\text{eff}}^2}$, and so:

$$n_{\text{eff}} = \frac{n_1\left({k_x}/{k_0}\right)}{\sqrt{n_1^2 - \left({k_x}/{k_0}\right)^2}} \qquad (9)$$

The refractive index values for Al_2O_3 and free-space at 540 nm are $n_2 = 1.7701$ and $n_3 = 1$, respectively. Using equation (9), Supplementary Table 4 shows excellent agreement with the expected values for n_{eff} in the limiting cases of $d = 0$ nm and $d \rightarrow \infty$. Using the experimental values for the wavelength shift and linewidths (from Figure 5c of the manuscript) and the data in Supplementary Table 4, the bulk sensitivity and FOM can be directly calculated.

d (nm)	$k_{1,z}/k_0$	$k_{2,z}/k_0$	$k_{3,z}/k_0$	k_x/k_0	$Real\{n_{\text{eff}}\}$
0	0.1226+3.4198i	1.4257	0.3223i	1.0506	1.0000
1	0.1225+3.4209i	1.4230	0.3338i	1.0542	1.0031
2	0.1225+3.4221i	1.4202	0.3456i	1.0580	1.0066
3	0.1224+3.4233i	1.4172	0.3577i	1.0620	1.0099
4	0.1224+3.4246i	1.4140	0.3700i	1.0662	1.0135
5	0.1223+3.4260i	1.4107	0.3825i	1.0706	1.0172
6	0.1223+3.4274i	1.4071	0.3954i	1.0753	1.0213
7	0.1222+3.4290i	1.4034	0.4086i	1.0802	1.0255
8	0.1222+3.4306i	1.3994	0.4219i	1.0853	1.0298
9	0.1221+3.4323i	1.3952	0.4356i	1.0907	1.0344
⋮	⋮	⋮	⋮	⋮	⋮
60	0.1131+3.7047i	0.0101	1.4616i	1.7709	1.5562
70	0.1118+3.7483i	0.5700i	1.5688i	1.8604	1.6159
⋮	⋮	⋮	⋮	⋮	⋮
$d \to \infty$	0.1081+3.8777i	1.1457i	1.8571i	2.1092	1.7709

Supplementary Table 4 | Effective refractive index of a bilayer dielectric medium surrounding a metal film. Effective index n_{eff} *vs*. dielectric layer thickness d (medium 2) for the three-layer system shown in Supplementary Figure 10a assuming medium 1 to be silver Ag, medium 2 to be Al_2O_3, and medium 3 to be free-space.

Supplementary Note 3 | Refractive index sensing:

In addition to demonstrating the versatility of the optimization algorithm, incorporating the interference model, to perform linewidth optimization necessary for sensing applications at any arbitrary wavelength and angle of incidence (Figure 5 of the manuscript), we summarize here the sensing capabilities of the multi-spectral response of an aperiodic device designed on Au-film for operation spanning the visible wavelengths. Simultaneous illumination of the sample at multiple angles of incidence would result in multiple discrete pre-defined spectral peaks in transmission thereby allowing for multiplexed sensing capabilities, which can result in higher-sensitivity than is possible from devices that exhibits only one spectral peak [12]. The aperiodic Au device was designed to fit within the same lateral foot-print as the Ag aperiodic slit-groove device (≤ 10 μm), and is theoretically implemented here to exhibit spectral peaks at 790 nm, 725 nm, 665 nm, 630 nm, and 560 nm for incident angles 0°, 5°, 10°, 15° and 20°, respectively, (Supplementary Figure 7). As the refractive index of the medium surrounding the patterned side of the device is varied from 1.00 to 1.02, each of the five-spectral peaks were found to red-shift with comparable index-sensitivities. Representative spectral-shifts as a function of change in refractive index for two spectral peaks corresponding to angles of incidence of θ = 5° and 15° are shown in Supplementary Figure 11a, and the device sensitivity response is plotted in Supplementary Figure 11b. For the Au multi-band device operating at θ = 0°, the bulk sensitivity is calculated to be S_b = 532 nm/RIU. Combining this with the resonance linewidth of $\Delta\lambda_{1/2}$ = 24 nm at λ_i = 615 nm gives a figure-of-merit, FOM = 22. For other angles of incidence, the FOM values are 35, 42, 25, and 25 at θ = 5°, 10°, 15° and 20°, respectively. These FOM values for the multiband structure over multiple angles of incidence are comparable to the typical values for plasmonic sensors [12-16].

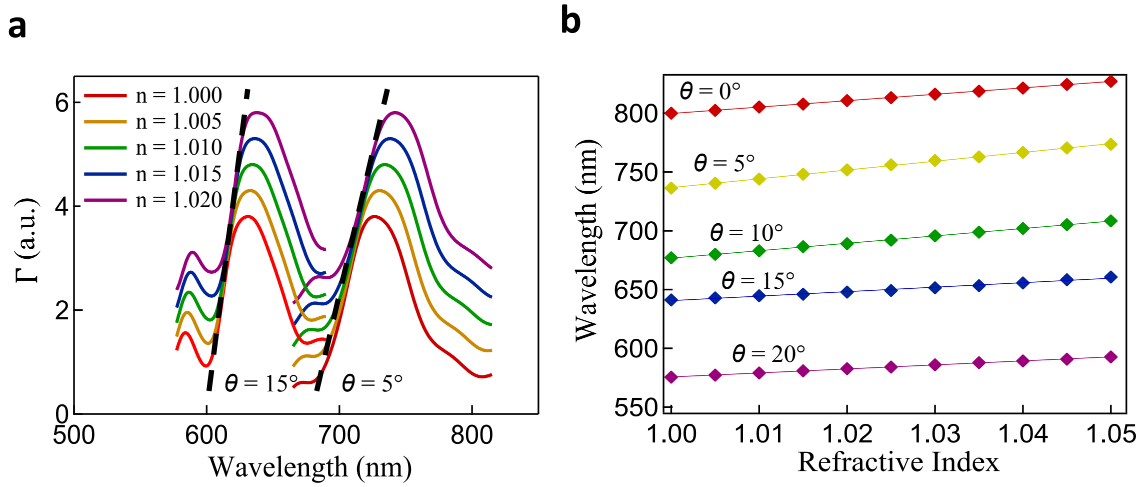

Supplementary Figure 11 | Multiplexed plasmonic sensing. a, Representative shift in spectral response of the five-peak Au device (shown in Supplementary Figure 7) as a function of change in refractive index of the surrounding media when illuminated at multiple angles of incidence (θ = 5° and 15°). The spectra in **a** are offset vertically for clarity. **b,** Summary of the shift in spectral peak as a function of change in refractive index for the Au slit-groove array devices at the five pre-defined angles of incidence. The slope of each curve corresponds to the index-sensitivity of the device which in conjunction with linewidth is used to calculate the FOM.

Supplementary Note 4 | SPP propagation length and Ag degradation:

The SPP propagation decay length L_{SPP} is experimentally measured (using the method described in ref. 17) to be ≈ 7 µm at λ_0 = 690 nm on an evaporated Ag-air interface used in the manuscript (Supplementary Figure 12, blue squares). For an equivalent Ag-air interface fabricated using the template-stripping approach, the SPP propagation decay length L_{SPP} is experimentally measured to be ≈ 30 µm at λ_0 = 690 nm on an evaporated Ag-air interface used in the manuscript (Supplementary Figure 12, purple spheres), a value that closely matches the theoretical SPP decay length calculated using the bulk effective permittivity of template-stripped Ag measured by a spectroscopic ellipsometer (Supplementary Figure 12, dashed black line).

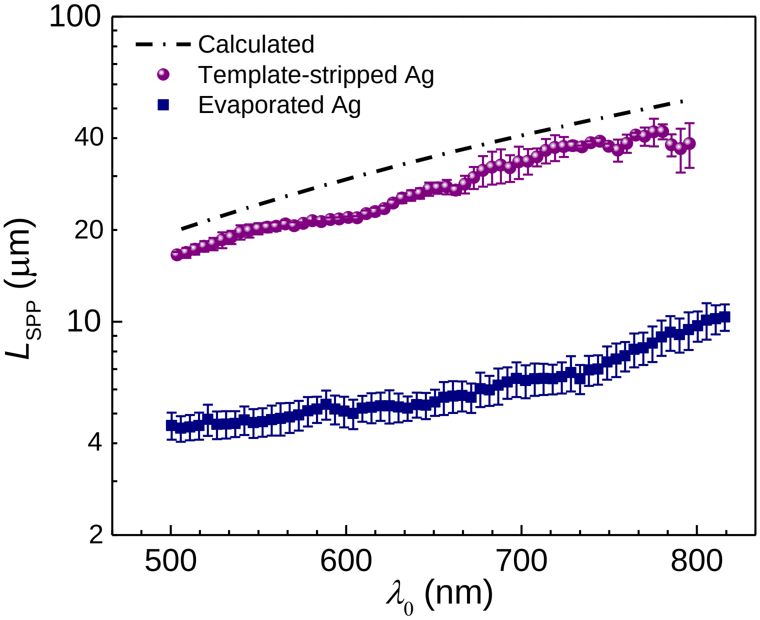

Supplementary Figure 12 | Propagation decay length of SPPs propagating on a Ag-air interface. Experimentally measured 1/e decay length L_{SPP} of SPPs for free-space wavelengths ranging from 500 nm to 800 nm on an evaporated Ag-air interface (blue squares) and a template-stripped Ag-air interface (purple spheres). The theoretical SPP decay length calculated using the bulk effective permittivity of template-stripped Ag (dashed black line).

Note that oxidation of Ag can also have a detrimental issue on device performance when operated under ambient conditions for long periods of time. We have not observed any degradation of Ag films used in our experiments as they were only exposed to air for the duration of the experiments (few minutes to an hour) and stored in a dry-environment. A few-nm thick atomic-layer-deposited protective overcoat of low-loss oxide (Al_2O_3) or use of doped-Ag films has been shown to dramatically improve the stability of Ag films without any compromise on the optical performance [18, 19].

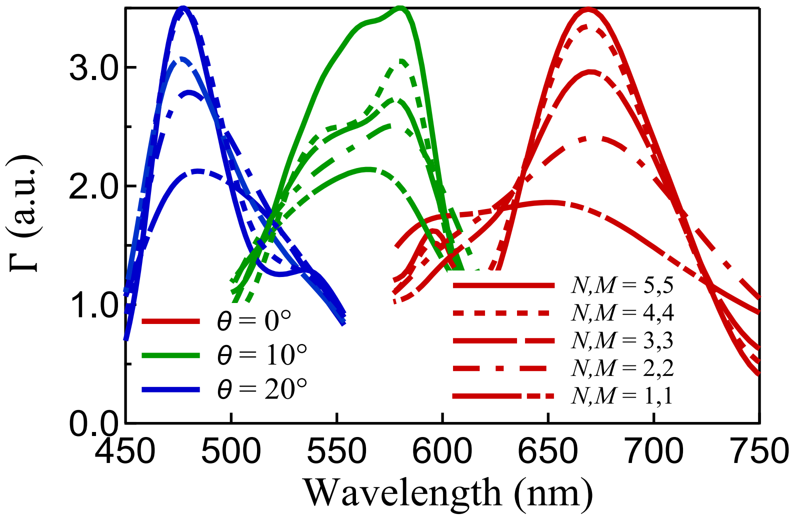

Supplementary Figure 13 | Number of Grooves Dependence. Optimized relative spectral transmission (Γ) through an aperiodic plasmonic device as a function of increasing number of grooves on both sides of the slit $(N, M = 1, 1)$ to $(N, M = 5, 5)$ for the three angles of incidence $\theta = 0°$, $10°$ and $20°$.

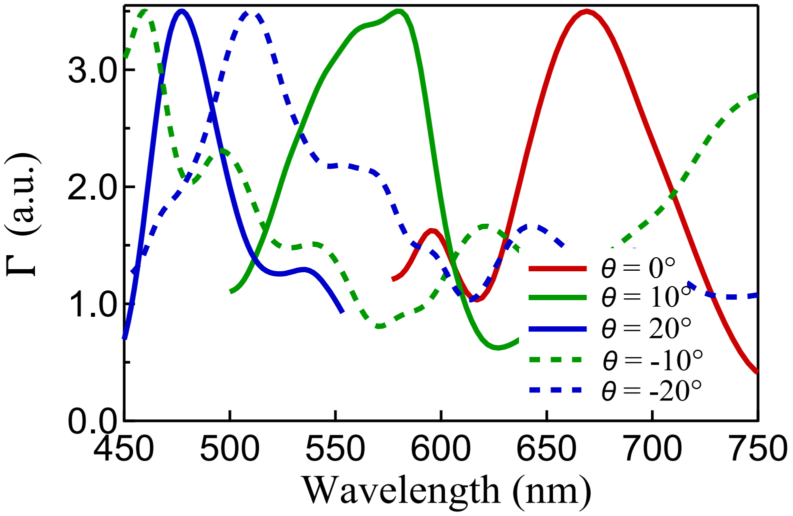

Supplementary Figure 14 | Relative transmission at off-angle incidence. Relative spectral transmission (Γ) through the aperiodic plasmonic device (Figure 2 of the manuscript) at non-optimized angles of incidence of $\theta = -10°$ and $-20°$ (dotted lines) along with the spectral transmission at optimized angles of $\theta = 0°$, $10°$ and $20°$ (solid lines).

Supplementary References:

[1] Burgos, S. P., Yokogawa, S. and Atwater, H. A. Colour imaging via nearest neighbor hole coupling in plasmonic colour filters integrated onto a complementary metal-oxide semiconductor image sensor. *ACS Nano* **7**, 10038–10047 (2013).

[2] Chen, Q., Chitnis, D., Walls, K., Drysdale, T. D., Collins, S. and Cumming, D. R. CMOS photodetectors integrated with plasmonic colour filters. *IEEE Photonics Technology Letters* **24**, 197–199 (2012).

[3] Zheng, B. Y., Wang, Y., Nordlander, P. and Halas, N. J. Colour-selective and CMOS-compatible photodetection based on Aluminum plasmonics. *Advanced Materials* **26**, 6318–6323 (2014).

[4] Yu, Y., Chen, Q., Wen, L., Hu, X. and Zhang, H. F. Spatial optical crosstalk in CMOS image sensors integrated with plasmonic colour filters. *Optics Express* **23**, 21994–22003 (2015).

[5] Agranov G., Berezin V. and Tsai R. H. Crosstalk and microlens study in a colour CMOS image sensor. *IEEE Trans. Electron. Devices* **50**, 4–11 (2003).

[6] Sounas, D. L. and Alù, A. Colour separation through spectrally-selective optical funneling. *ACS Photonics* **3**, 620−626 (2016).

[7] Li, E., Chong, X., Ren, F. and Wang, A. X. Broadband on-chip near-infrared spectroscopy based on a plasmonic grating filter array. *Optics Letters* **41**, 1913–1916 (2016).

[8] Haïdar, R., Vincent, G. G., Collin, S. P., Bardou, N., Guérineau, N., Deschamps, J. L. and Pelouard, J. L. Free-standing subwavelength metallic gratings for snapshot multispectral imaging. *Applied Physics Letters* **96**, 221104 (2010).

[9] Maier, S. A. *Plasmonics: Fundamentals and Applications*. (Springer US, 2007).

[10] Palik, E. *Handbook of optical constants of solids.* (Academic Press, New York, 1985).

[11] Raether, H. *Surface-plasmons on smooth and rough surfaces and on gratings*. *Springer Tracts in. Mod. Phys.* **111**, 1–133 (Springer, 1988).

[12] Gao, Y., Xin, Z., Gan, Q., Cheng, X. and Bartoli, F. J. Plasmonic interferometers for label-free multiplexed sensing. *Optics Express* **21**, 5859–5871 (2013).

[13] Shen, Y., Zhou, J., Liu, T., Tao, Y., Jiang, R., Liu, M., Xiao, G., Zhu, J., Zhou, Z. -K., Wang, X., Jin, C. and Wang, J. Plasmonic gold mushroom arrays with refractive index sensing figures of merit approaching the theoretical limit. *Nature Communications* **4**, 2381 (2013).

[14] Feng, J., Siu, V. S., Roelke, A., Mehta, V., Rhieu, S. Y., Palmore, G. T. R. and Pacifici, D. Nanoscale plasmonic interferometers for multispectral, high-throughput biochemical sensing. *Nano Letters* **12**, 602–609 (2012).

[15] Valsecchi, C. and Brolo, A. G. Periodic metallic nanostructures as plasmonic chemical sensors. *Langmuir* **29**, 5638–5649 (2013).

[16] Wang, X. -Y., Wang, Y. -L., Wang, S., Li, B., Zhang, X. -W., Dai, L. and Ma, R. -M. Lasing enhanced surface plasmon resonance sensing. *Nanophotonics* **5**, 52–58 (2016).

[17] Nagpal, P. Lindquist, N. C. Oh, S.-H. and Norris, D. J. Ultrasmooth patterned metals for plasmonics and metamaterials. *Science* **325**, 594–597 (2009).

[18] Zhang, X., Zhao, J., Whitney, A. V., Elam, J. W. and Van Duyne, R. P. Ultrastable substrates for surface-enhanced Raman spectroscopy: Al_2O_3 overlayers fabricated by atomic layer deposition yield improved Anthrax biomarker detection. *Journal of the American Chemical Society* **128,** 10304–10309 (2006).

[19] Zhang, C. *et al*. An Ultrathin, smooth, and low-loss Al-doped Ag film and its application as a transparent electrode in organic photovoltaics. *Advanced Materials* **26,** 5696–5701 (2014).
